# Deubiquitinase USP7-mediated MCL-1 up-regulation enhances Arsenic and Benzo(a)pyrene co-exposure-induced Cancer Stem Cell-like property and Tumorigenesis

**DOI:** 10.7150/thno.47897

**Published:** 2020-07-11

**Authors:** Ping Yang, Jie Xie, Yunfei Li, Hsuan-Pei Lin, William Fenske, Marco Clementino, Yiguo Jiang, Chengfeng Yang, Zhishan Wang

**Affiliations:** 1 Department of Toxicology and Cancer Biology, University of Kentucky College of Medicine, Lexington, Kentucky, USA.; 2 School of Public Health, Guangzhou Medical University, Guangzhou, Guangdong, P.R. China.; 3 School of Health Sciences, Wuhan University, Wuhan, Hubei, P.R. China.

**Keywords:** USP7, MCL-1, arsenic, benzo(a)pyrene, cancer stem cell-like property

## Abstract

**Rationale:** MCL-1 is up-regulated in cancer and a target for cancer treatment. How MCL-1 is up-regulated and whether MCL-1 up-regulation plays a role in tumorigenic process is not well-known. Arsenic and benzo(a)pyrene (BaP) are well-recognized lung carcinogens and we recently reported that arsenic and BaP co-exposure acts synergistically in inducing cancer stem cell (CSC)-like property and lung tumorigenesis. This study was performed to further investigate the underlying mechanism focusing on the role of MCL-1.

**Methods:** The spheroid formation assay and nude mouse tumorigenesis assay were used to determine the CSC-like property and tumorigenicity of arsenic plus BaP co-exposure-transformed human bronchial epithelial BEAS-2B cells, respectively. Biochemical, pharmacological and genetic approaches were used to manipulate gene expressions, dissect signaling pathways and determine protein-protein interactions. Both loss-of-function and gain-of-function approaches were used to validate the role of MCL-1 in arsenic plus BaP co-exposure-enhanced CSC-like property and tumorigenicity.

**Results:** Arsenic plus BaP co-exposure-transformed cells express significantly higher protein levels of MCL-1 than the passage-matched control, arsenic or BaP exposure alone-transformed cells. Knocking down MCL-1 levels in arsenic plus BaP co-exposure-transformed cells significantly reduced their apoptosis resistance, CSC-like property and tumorigenicity in mice. Mechanistic studies revealed that arsenic plus BaP co-exposure up-regulates MCL-1 protein levels by synergistically activating the PI3K/Akt/mTOR pathway to increase the level of a deubiquitinase USP7, which in turn reduces the level of MCL-1 protein ubiquitination and prevents its subsequent proteasome degradation.

**Conclusions:** The deubiquitinase USP7-mediated MCL-1 up-regulation enhances arsenic and BaP co-exposure-induced CSC-like property and tumorigenesis, providing the first evidence demonstrating that USP7 stabilizes MCL-1 protein during the tumorigenic process.

## Introduction

Apoptosis resistance or evasion of cell death is a hallmark of cancer 1. Apoptotic cell death is mediated by either the intrinsic or the extrinsic apoptotic pathway. The intrinsic apoptotic pathway is largely regulated by the pro- and anti-apoptotic members of the B cell lymphoma 2 (BCL-2) family 2, 3. Myeloid cell leukemia-1 (MCL-1) is an anti-apoptotic member of the BCL-2 family, playing a critical role in apoptosis resistance. MCL-1 protein level up-regulation has been found in multiple types of cancer and has been proposed as a target for cancer treatment 4, 5. However, little is known about the mechanism of how MCL-1 expression level is up-regulated and whether MCL-1 up-regulation plays an important role during tumor development process.

MCL-1 is unique in the BCL-2 family in that its protein has a short half-life due to its fast turnover by ubiquitination and proteasome degradation 6. Studies have shown that inhibition or depletion of deubiquitinases USP9X or USP13 significantly reduces MCL-1 protein levels and cancer cell survival as well 5, 7. Identification of additional deubiquitinases that regulate MCL-1 protein stability may offer alternative targets for cancer prevention and treatment.

Both arsenic and benzo[a]pyrene (BaP) are classified as Group I carcinogens causing lung cancer and other types of cancer in humans 8, 9. Arsenic is a widely distributing and naturally occurring chemical and arsenic-contaminated drinking water is the main source of general population arsenic exposure 8. BaP is produced when organic matters are incompletely combusted 9. Well-done barbecued-meat and cigarette smoke usually contain high levels of BaP and are the common sources of human BaP exposure 10, 11. Due to the wide presences of arsenic and BaP in environment, water and food, arsenic and BaP co-exposure is common in humans. However, the adverse health effects resulting from arsenic and BaP co-exposure and the underlying mechanisms have not been well understood. Some early studies showed that arsenic and BaP co-exposure via intratracheal instillation significantly increases the incidence of lung cancer in rats and hamsters 12, 13. Moreover, our recent study showed that arsenic exposure through drinking water in conjunction with BaP exposure via oral gavage significantly increases lung tumor multiplicity and tumor burden in mice 14. Together, these findings indicate that arsenic and BaP co-exposure via inhalation or oral ingestion significantly increases lung cancer risk and tumor burden. It is thus imperative to investigate the mechanism of how arsenic and BaP co-exposure enhances lung carcinogenesis. A better understanding on the mechanism of how arsenic and BaP co-exposure increases lung carcinogenesis may identify molecular targets for prevention and treatment of lung cancer resulting from arsenic and BaP co-exposure.

In this study, we demonstrated that MCL-1 protein level is up-regulated in arsenic and BaP co-exposure-transformed cells, which plays a critical role in arsenic and BaP co-exposure-induced CSC-like property and tumorigenesis. Moreover, we further determined that MCL-1 up-regulation is achieved by the increase of its protein stability through the deubiquitinase USP7-mediated deubiquitination. This is the first study reporting that the anti-apoptotic MCL-1 protein stability is regulated by the deubiquitinase USP7 in tumor cells (arsenic and BaP co-exposure-transformed cells). The findings from this study not only provide a new mechanism for understanding the synergistic tumorigenic effect of arsenic and BaP co-exposure, but also reveal an important role and a new mechanism for MCL-1 protein up-regulation in the tumorigenic process.

## Materials and Methods

### Cell culture and reagents

Immortalized human bronchial epithelial BEAS-2B cells were purchased from American Type Culture Collection (ATCC, Manassas, VA) and are mycoplasma-free. Immortalized human bronchial epithelial 16HBE cells were obtained from Dr. Dieter C Gruenert (University of California, San Francisco) and are mycoplasma-free. Passage-matched BEAS-2B control cells (BEAS-2B-Control), arsenic exposure alone-transformed cells (BEAS-2B-As), BaP exposure alone-transformed cells (BEAS-2B-BaP) and arsenic plus BaP co-exposure-transformed cells (BEAS-2B-As+BaP) were generated, characterized and reported in our recent publication 14. Briefly, BEAS-2B cells were continuously exposed to a vehicle control (dimethyl sulfoxide, DMSO), arsenic (NaAsO_2_, 1 μM, Sigma), BaP (2.5 μM, Sigma) or arsenic (NaAsO_2_, 1 μM) plus BaP (2.5 μM) for 30 weeks. For 16HBE cell transformation experiment, 16HBE cells were continuously exposed to the same concentrations of vehicle control DMO, arsenic, BaP alone or in combination for 40 weeks. All BEAS-2B cells were cultured in Dulbecco's Modified Eagle Medium (DMEM) supplemented with 5% fetal bovine serum (FBS) at 37 °C in a humidified 5% CO_2_ atmosphere. All 16HBE cells were cultured in minimal essential medium (MEM) supplemented with 10% FBS. ABT-737 (#11501), wortmannin (#10010591), rapamycin (#13346), cycloheximide (Chx) (#14126), and MG132 (#10012628) were purchased from Cayman Chemical company (Cayman Chemical, MI, USA).

### Western blot analysis

At the end of various treatments, cells were collected and lysed using Tris-sodium dodecyl sulfate (SDS) cellular lysis buffer and subjected to SDS-polyacrylamide gel electrophoresis (PAGE) analysis as described in our previous publication 15. The following primary antibodies were used: anti-MCL-1 (#94296), anti-BCL-XL (#2764), anti-BCL-2 (#4223), anti-Puma (#24633), anti-Bax (#5023), anti-Bim (#2819), anti-PARP (#9542), anti-cleaved PARP (#5625), anti-caspase3 (#9662), anti-cleaved caspase 3 (#9664), anti-USP7 (#4833), anti-phospho-mTOR (Ser2448) (#5536), anti-phospho-p70 S6 Kinase (Thr389) (9234), anti-phospho-4EBP1 (Thr37/46) (#2855), anti-4EBP1 (#9644), anti-Akt (#9272), anti-phospho-Akt (Ser473) (#4060), anti-Ubiquitin (#3936), anti-Ki67 (#9449) (Cell Signaling Technology, Beverly, MA), anti-β-actin (#A5441) (Sigma).

### Flow cytometry analysis of apoptosis

Briefly, 3×10^6^ cells were plated, cultured for overnight and then treated with 20 µM of ABT-737 for 24 h. Apoptosis was detected using APC-conjugated Annexin V and 7-Amin-Actinomycin D (7-AAD) (BD Biosciences, USA). At the end of ABT-737 treatment, cells were collected and washed twice in cold PBS, re-suspended in Annexin V‑binding buffer (#51-66121E, BD Biosciences, USA) at a concentration of 1×10^6^ cells/ml. One hundred μl of the cell suspension was incubated with 5 μl of APC Annexin V (#550475, BD Biosciences, USA) and 5 μl of 7-AAD solution (#51-68981E, BD Biosciences, USA) in dark at room temperature for 15 minutes. Following the addition of 400 μl binding buffer to each cellular sample at the end of incubation, cells were analyzed using FACSAria™ III Cell Sorter flow cytometer (BD Biosciences, USA). The flow cytometry results were analyzed using BD FlowJo software (BD Biosciences, USA).

### RNA interference

Negative control small interfering RNA (siRNA) and ON-TARGETplus SMARTpool siRNA (a mixture of 4 specific target siRNAs) for MCL-1 and BCL-XL were purchased from Thermo Scientific Dharmacon (Lafayette, CO). Control, MCL-1 or BCL-XL targeting siRNA duplexes (100 nM) were transfected into arsenic and BaP co-exposure-transformed cells (BEAS-2B-As+BaP) using Lipofectamine 2000 (Invitrogen, Carlsbad, CA, USA) in serum-free medium following the manufacturer's instructions. 48 h after transfection, cells were treated with 20 µM of ABT-737 for 24 h, and cells were collected for Western blot analysis. Successful knockdown of MCL-1 and BCL-XL was confirmed by Western blot.

### Clonogenic assay

The clonogenic assay was performed following the protocol described in our recent publication 16. Briefly, cells were seeded onto 60-mm culture dishes at a density of 100 cells per dish. After 48 h culture, cells were treated with 10 µM of ABT-737 for 48 h. Cells were then washed with PBS twice and cultured in drug-free media for additional 11 days. At the end of the culture, cell clones were stained with 0.01% (w/v, in 4% paraformaldehyde) crystal violet, photographed and counted.

### Quantitative PCR (q-PCR) analysis

Cellular total RNAs were extracted using the Trizol reagent (Invitrogen) and RNA concentrations were measured using NanoDrop^TM^ One (Thermo Scientific). The cDNA was synthesized using the SuperScriptTM II RT (Invitrogen) for q-PCR amplification, which was carried out in ABI QuantStudio 3 System using ABI TaqMan gene expression assay. The 18S RNA level was analyzed and used to normalize specific gene mRNA expression levels as described in our previous studies 15, 17.

### Immunoprecipitation (IP) analysis

The IP analysis was performed as described in our previous publications 17, 18. Briefly, cells were lysed with a lysis buffer containing 50 mM Tris, pH 7.5, 150 mM NaCl, 0.5% NP-40, 1 mM phenylmethylsulfonyl fluoride for 10 minutes on ice and were then centrifuged at 13,000 g for 10 minutes. The supernatants were collected and incubated with an anti-MCL-1 antibody overnight at 4 °C, followed by incubation with protein PLUS-A/G agarose beads (Santa Cruz Biotechnology) at 4 °C for 2 h. Thereafter, the beads were spun down and washed three times with the cell lysis buffer, and the immunoprecipitated proteins were analyzed by Western blot.

### Generation of MCL-1, Myr-Akt1 stable overexpression cells

To generate MCL-1 and the constitutively active Akt (Myr-Akt1) overexpression cells, human MCL-1 full-length cDNA (Addgene #117729) and Myr-Akt1 cDNA (Addgene #53583) were cloned into the pLenti6.3 vector (Invitrogen) or the pLenti7.3 vector (Invitrogen) following the manufacturer's instructions, respectively. Vector control and MCL-1-expressing and Myr-Akt1-expressing lentiviral particles were packaged as described in our recent studies 18, 19. To establish vector control MCL-1 or Myr-Akt1 overexpression cells, BEAS-2B-As+BaP cells were transduced with vector control (pLenti6.3 or pLenti7.3), MCL-1-expressing (pLenti6.3-MCL-1) or Myr-Akt1-expresing (pLenti7.3-Myr-Akt1) lentiviral particles, respectively, and selected with blasticidin (for pLenti6.3 vector) or via flow cytometry sorting of EGFP-positive cells. MCL-1 or Myr-Akt1 overexpression was confirmed by Western blot.

### Generation of MCL-1 stable knockdown cells

The vector control and MCL-1 stable knockdown cells were generated by transducing the BEAS-2B-As+BaP cells with the non-targeting control small hairpin RNA (shRNA) lentiviral (pZIP-hCMV-ZsGreen-Puro-Control shRNA) or MCL-1 targeting shRNA lentiviral (pZIP-hCMV-ZsGreen-Puro-MCL-1 shRNA) particles (Transomic Technologies, AL). As described in our previous publication 14, 20, cells were selected with puromycin (1 μg/ml) 48 h after the lentiviral particle transduction. Specific knockdown of MCL-1 was confirmed by Western blot analysis.

### Serum-free suspension culture spheroid formation assay

The serum-free suspension culture spheroid formation assay reflecting the stem cell property was performed following the published protocol 14, 18, 21, 22. Briefly, single cells were plated in ultralow attachment 24-well culture plates (Corning, Corning, NY) at a density of 2.5×10^3^ cells per well in serum-free DMEM. The culture media was supplemented with human recombinant basic fibroblast growth factor (bFGF, 20 ng/ml), human recombinant epidermal growth factor (EGF, 20 ng/ml) (R&D, Minneapolis, MN), B27 (50 times diluted from the original 50× stock solution, Invitrogen, Carlsbad, CA) and heparin (4 μg/ml, Sigma). Plates were incubated at 37 °C in a humidified 5% CO_2_ atmosphere for 10 days. At the end of incubation, spheres were viewed, photographed and counted (if > 100 μm) under a phase-contrast microscope.

### ALDEFLUOR assay

The ALDEFLUOR assay is another commonly used assay for analyzing CSC property. CSCs have high aldehyde dehydrogenase (ALDH) activity and the ALDEFLUOR assay detects the percentage of cells with high ALDH activity 23. The ALDEFLUOR assay was performed as described in our recent publication 14. The ALDH inhibitor DEAB treatment was used to verify that the gated ALDEFLUOR positive cells are diminished upon DEAB treatment and thus indeed ALDEFLUOR positive cells.

### Nude mouse xenograft tumorigenesis study

The animal protocol was reviewed and approved by the University of Kentucky Institutional Animal Care and Use Committee. Seven-week-old female nude mice (Nu/Nu, Charles River Laboratory) were randomly divided into 2 groups with 10 mice in each group. Arsenic and BaP co-exposure-transformed cells with MCL-1 stably knocked down (BEAS-2B-As+BaP-MCL1 shRNA) or control shRNA (BEAS-2B-As+BaP-Control shRNA) (0.25×10^6^ cells in 0.1 ml of 1:1 growth factor-reduced matrigel and PBS) were injected subcutaneously into both flanks of female nude mice (10 mice in each group). After cell injections, nude mice were maintained under specific pathogen-free conditions and monitored weekly. All mice were euthanized 12 weeks after the injection, and the xenograft tumor tissues were harvested and fixed with 10% formalin solution for histology analysis.

### Mouse xenograft tumor tissue H&E and Ki-67 immunohistochemistry (IHC) staining and mouse lung tumor MCL-1 immunofluorescence (IF) staining

Nude mouse xenograft tumor tissue sections were prepared and subjected to H&E and Ki-67 IHC staining as described previously 19, 24. Mouse lung normal and tumor tissues resulting from exposure to BaP alone or arsenic plus BaP in our recent study 14 were sectioned and used for MCL-1 IF staining following our published protocols 19, 20. The IHC and IF staining images were taken using Nikon NIS-Elements software.

### Statistical analysis

The statistical analyses for the significance of differences in presented-numerical data (mean ± SD) were carried out by testing different treatment effects using two-tailed t-tests for comparison of two data sets or one-way analysis of variance (ANOVA) for multiple data sets. The significance of the difference of tumor incidence rate in nude mice injected with the vector control and MCL-1 stable knockdown cells was tested using the Fisher's exact test. A *p* value of <0.05 was considered statistically significant.

## Results

### MCL-1 is up-regulated and mediates apoptosis resistance in arsenic and BaP co-exposure-transformed cells

**Figure 1:**
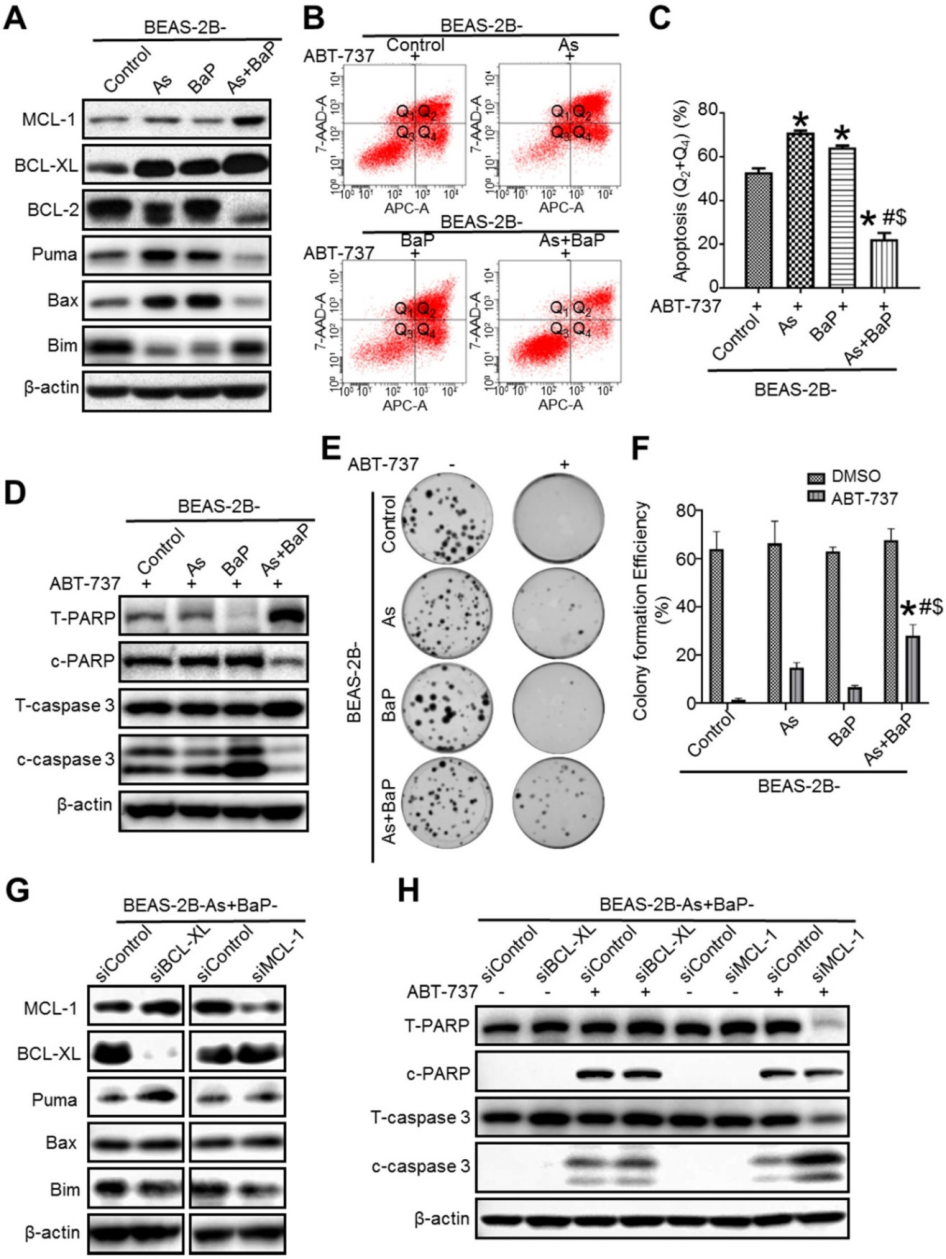
MCL-1 is up-regulated in arsenic and BaP co-exposure transformed cells mediating apoptosis resistance. **A.** Representative Western blot analysis of the levels of anti-apoptotic proteins MCL-1, BCL-XL, BCL-2 and pro-apoptosis proteins Puma, Bax and Bim in passage-matched control cells (BEAS-2B-Control), arsenic exposure alone-transformed cells (BEAS-2B-As), BaP exposure alone-transformed cells (BEAS-2B-BaP) and arsenic plus BaP co-exposure-transformed cells (BEAS-2B-As+BaP). **B-D.** Apoptosis analysis in BEAS-2B-Control, BEAS-2B-As, BEAS-2B-BaP and BEAS-2B-As+BaP cells treated with 20 µM of ABT-737 for 24 h. Representative histograms of flow cytometry analysis of apoptosis by Annexin V staining (**B**). Q1, Q2, Q3, Q4 indicate necrocytosis, late apoptosis cells, survival cells, and early apoptosis, respectively. Summarized results of flow cytometry analysis of apoptosis (**C**) (mean ± SD, n=3). *p<0.05, compared to the BEAS-2B-Control group; # *p*<0.05, compared to the BEAS-2B-As group; $ *p*<0.05, compared to the BEAS-2B-BaP group. Representative Western blot analysis of total and cleaved PARP and caspase-3 protein levels in cells treated with ABT-737 (**D**). **E-F.** Representative clonogenic assay images (E) and summarized clonogenic assay results (F) (mean ± SD, n=3) of cells treated with 10 µM of ABT-737 or a vehicle control DMSO for 48 h and cultured for additional 11 days. * *p*<0.05, compared to ABT-737-treated BEAS-2B-Control cells; # *p*<0.05, compared to ABT-737-treated BEAS-2B-As cells; $ *p*<0.05, compared to ABT-737-treated BEAS-2B-BaP cells. **G.** Representative Western blot analysis of MCL-1, BCL-XL, Puma, Bax and Bim protein levels in BEAS-2B-As+BaP cells transfected with Control siRNA (siControl), BCL-XL siRNA (siBCL-XL) or MCL-1 siRNA (siMCL-1). **H.** Representative Western blot analysis of total and cleaved PARP and caspase-3 protein levels in BEAS-2B-As+BaP cells transfected with Control siRNA (siControl), BCL-XL siRNA (siBCL-XL) or MCL-1 siRNA (siMCL-1) and treated with a vehicle control or 20 µM of ABT-737 for 24 h. Similar results were obtained in the repeated experiments.

Our recent study showed that arsenic and BaP co-exposure causes a significantly stronger effect in activating Akt and promoting cell transformation, CSC-like property and tumorigenesis, compared to arsenic or BaP exposure alone 14. Akt activation causes inhibition of the intrinsic apoptotic program via regulating the BCL-2 family protein levels 25. Since the intrinsic apoptosis is considered as a natural barrier to carcinogenesis and apoptosis resistance is a hallmark of cancer 1, 3, we sought to determine whether arsenic and BaP co-exposure-transformed cells display apoptosis resistance and the underlying mechanism. We first analyzed BCL-2 family several important anti- and pro-apoptotic protein levels. It was found that arsenic and BaP co-exposure-transformed BEAS-2B cells have significantly higher levels of anti-apoptotic protein MCL-1 and BCL-XL, but lower levels of pro-apoptotic protein Puma and Bax, compared to the passage-matched control cells as well as arsenic (As) or BaP exposure alone-transformed cells (Figure 1A). Previously, we also performed cell transformation experiment using another immortalized human bronchial epithelial 16HBE cells. It was found that arsenic and BaP co-exposure also synergizes in inducing 16HBE cell transformation as evidenced by forming significantly more soft agar colonies than arsenic or BaP exposure alone (Figure S1A). Similarly, the highest MCL-1 and BCL-XL protein levels are also detected in arsenic and BaP co-exposure-transformed 16HBE cells (Figure S1B). Moreover, immunofluorescence staining of MCL-1 revealed that MCL-1 levels are significantly higher in arsenic plus BaP co-exposure-induced mouse lung tumor tissues than mouse normal lung tissues or BaP exposure alone-induced mouse lung tumor tissues (Figure S1C). BaP exposure alone- and arsenic plus BaP co-exposure-induced mouse lung tumor formation was reported in our recent publication 14. These results suggest that arsenic and BaP co-exposure-transformed cells may display resistance to the intrinsic apoptotic program.

Next, we treated our passage-matched control cells, arsenic or BaP exposure alone-transformed cells, and arsenic and BaP co-exposure-transformed BEAS-2B cells with ABT-737, which is one of BCL-2 homology 3 (BH3) mimetics and is capable of binding to anti-apoptotic protein BCL-2, BCL-XL and BCLW to enhance the intrinsic apoptotic cell death 26, 27. The occurrence of apoptosis was determined using a flow cytometry-based analysis of Annexin V-positive cells. It was found that control cells, and arsenic or BaP exposure alone-transformed cells are highly sensitive to ABT-737 treatment-induced apoptosis (Figure 1B-C). In contrast, arsenic and BaP co-exposure-transformed cells are the least sensitive and highly resistant to ABT-737 treatment-induced apoptosis (Figure 1B-C). Further apoptosis analysis by Western blot revealed that arsenic and BaP co-exposure-transformed cells have the lowest protein levels of the cleaved poly (ADP-ribose) polymerase (c-PARP) and caspase-3 (c-caspase-3) in response to ABT-737 treatment (Figure 1D). Moreover, the clonogenic assay showed that arsenic and BaP co-exposure-transformed cells treated with ABT-737 grow significantly more clones than ABT-737-treated control cells, and arsenic or BaP exposure-alone transformed cells (Figure 1E-F). Similarly, Western blot analysis also showed that arsenic and BaP co-exposure-transformed 16HBE cells have the lowest protein levels of the cleaved poly (ADP-ribose) polymerase (c-PARP) and caspase-3 (c-caspase-3) in response to ABT-737 treatment (Figure S1D). Together, these results demonstrate that arsenic and BaP co-exposure-transformed cells display a strong resistance to ABT-737 treatment-induced apoptotic cell death program.

Next, we began to determine the mechanism of how arsenic and BaP co-exposure-transformed cells obtained apoptosis resistance. Since the anti-apoptotic protein levels of MCL-1 and BCL-XL were greatly increased in the co-exposure-transformed cells (Figure 1A), we used siRNAs to specifically knock down MCL-1 or BCL-XL (Figure 1G), respectively. Interestingly, knocking down BCL-XL did not have a significantly stronger effect on ABT-737 treatment-induced apoptosis as determined by Western blot analysis of cleaved PARP and caspase-3 levels, compared to the control siRNA (Figure 1H). In contrast, compared to control siRNA, knocking down MCL-1 greatly increased ABT-737 treatment-induced apoptosis as evidenced by the significantly higher level of cleaved caspase-3 and the diminished level of total caspase-3 (Figure 1H). Importantly, although the cleaved PARP levels in MCL-1-knocked down and ABT-737-treated cells was not higher than the control siRNA-transfected and ABT-737-treated cells, the level of total PARP in MCL-1-knocked down cells was undetectable, indicating significantly more PARP cleavage and apoptosis (Figure 1H). In addition, although the levels of the pro-apoptotic protein Puma and Bax were greatly reduced in arsenic and BaP co-exposure-transformed cells (Figure 1A), knocking down MCL-1 level did not increase the levels of these pro-apoptotic proteins (Figure 1G). Together, these results suggest that the apoptosis resistance in arsenic and BaP co-exposure-transformed cells is mainly mediated by the up-regulation of the anti-apoptotic protein MCL-1.

### MCL-1 protein stability is significantly increased in arsenic and BaP co-exposure-transformation cells

**Figure 2:**
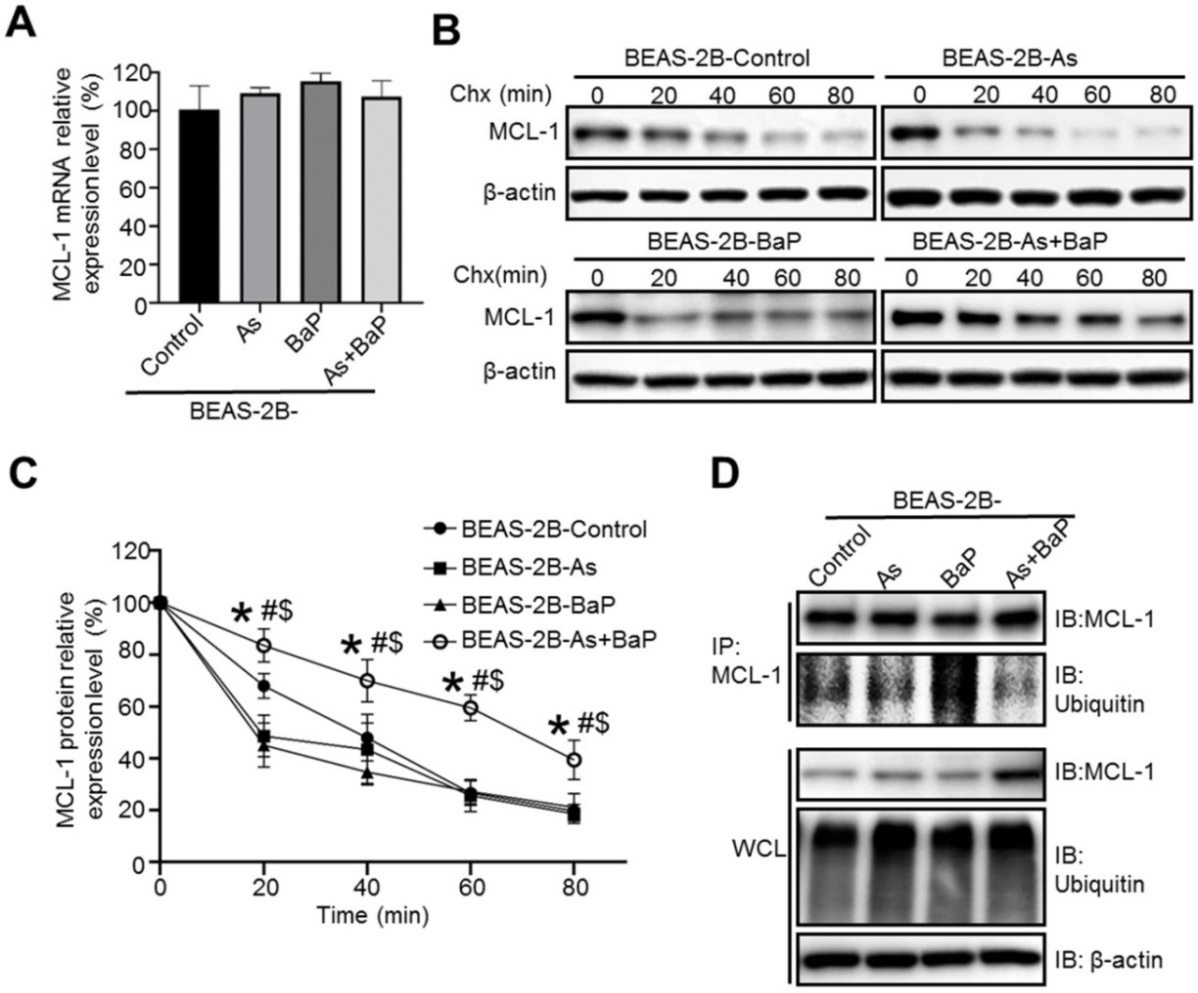
MCL-1 protein stability is significantly increased in arsenic and BaP co-exposure-transformation cells. **A.** Q-PCR analysis of MCL-1 mRNA expression levels in the BEAS-2B-Control, BEAS-2B-As, BEAS-2B-BaP and BEAS-2B-As+BaP cells (mean ± SD, n=3). **B-C.** Representative Western blot analysis (**B**) and the quantitated results (mean ± SD, n=3) (**C**) of MCL-1 protein half-life in BEAS-2B-Control, BEAS-2B-As, BEAS-2B-BaP and BEAS-2B-As+BaP cells treated with 5 µM of cycloheximide (Chx) for 0, 20, 40, 60, 80 minutes, respectively. * *p*<0.05, compared to the BEAS-2B-Control group; # *p*<0.05, compared to the BEAS-2B-As group; $ *p*<0.05, compared to the BEAS-2B-BaP group. **D.** Representative Western blot analysis of the MCL-1 IP experiment. IP: immunoprecipitation; IB: immunoblotting; WCL: whole cell lysate. Similar results were obtained in the repeated experiments.

Next, we sought to investigate the mechanism of MCL-1 up-regulation in arsenic and BaP co-exposure-transformed cells. We first performed q-PCR analysis of MCL-1 mRNA levels and found no significant differences among the four treatment groups of cells (Figure 2A), indicating that up-regulation of MCL-1 protein level is not due to the increased MCL-1 gene transcription.

We then determined whether MCL-1 up-regulation in the co-exposure-transformed cells is due to the increased MCL-1 protein stability. Cycloheximide (Chx) is a chemical inhibitor widely used to block protein synthesis by interfering with translational elongation machinery, enabling determination of a protein half-life 28.

As shown in Figure 2B-C, the half-life of MCL-1 protein in Chx-treated co-exposure-transformed cells was significantly extended, compared to MCL-1 protein half-life in Chx-treated control cells, arsenic or BaP exposure alone-transformed cells. The increased protein half-life usually results from the reduced protein ubiquitination and subsequent proteasome degradation. We further performed immunoprecipitation (IP) experiments to determine and compare MCL-1 ubiquitination levels among four treatment groups of cells. As shown in Figure 1D, the MCL-1 protein ubiquitination level was the lowest in co-exposure-transformed cells, compared to control, arsenic or BaP exposure alone-transformed cells. Interestingly, no significant differences of total protein ubiquitination levels in the whole cell lysate (WCL) of the four treatment groups of cells were observed (Figure 2D). Together, these results suggest that up-regulation of MCL-1 protein level in co-exposure-transformed cells is likely due to the increased MCL-1 protein stability resulting from the reduced MCL-1 protein ubiquitination.

### Inhibition of the phosphatidylinositol-3 kinase (PI3K)/AKT pathway significantly reduces MCL-1 protein stability in arsenic and BaP co-exposure-transformed cells and reverses their apoptosis resistance

**Figure 3:**
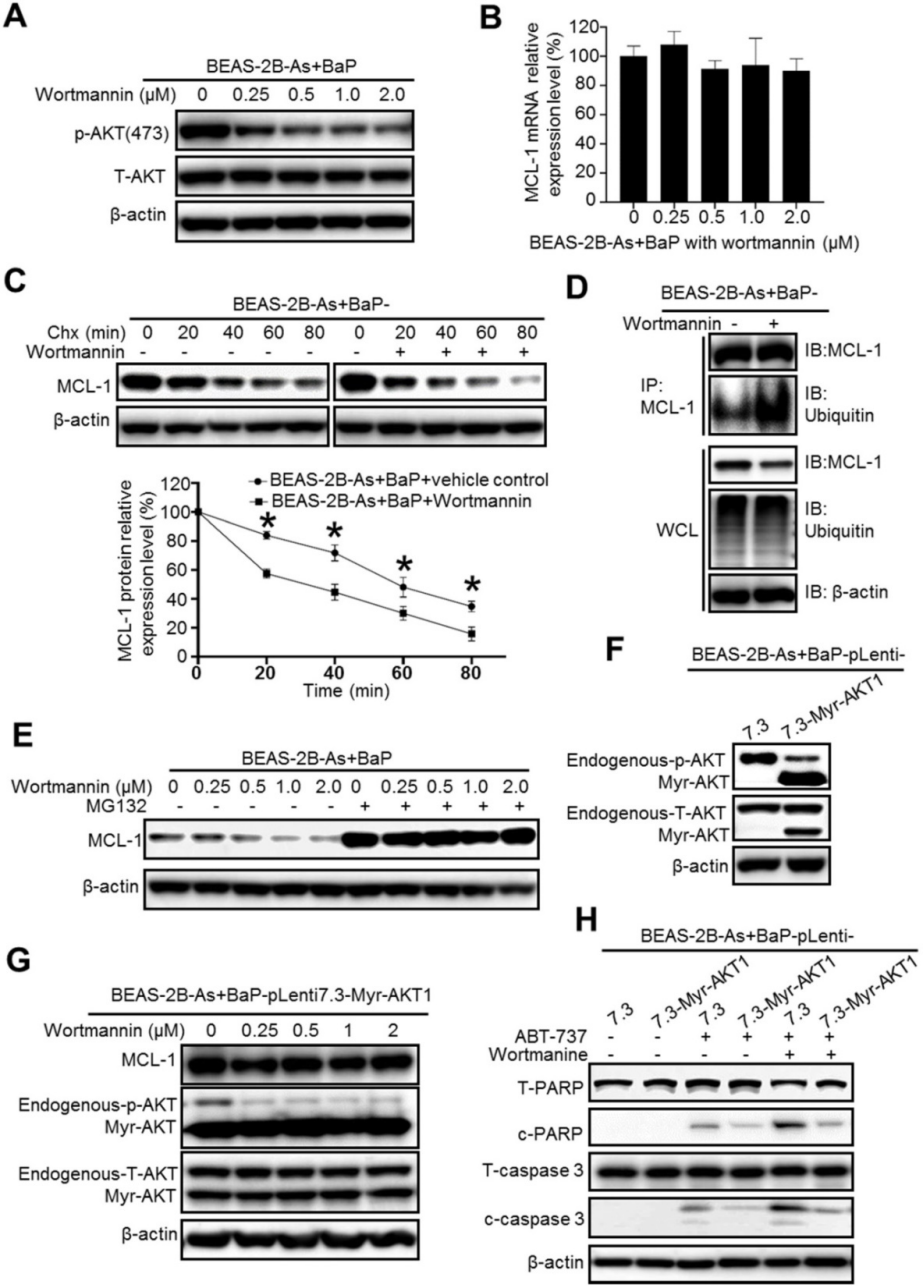
Inhibition of the PI3K/AKT pathway significantly reduces MCL-1 protein stability in arsenic and BaP co-exposure-transformed cells and reverses their apoptosis resistance. **A.** Representative Western blot analysis of phosphor- and total Akt level in BEAS-2B-As+BaP cells treated with a vehicle control or Wortmannin (0.25, 0.5, 1.0 or 2.0 µM) for 24 h. **B.** Q-PCR analysis of MCL-1 mRNA expression levels in BEAS-2B-As+BaP cells treated with a vehicle control or Wortmannin (0.25, 0.5, 1.0 or 2.0 µM) for 24 h (mean ± SD, n=3). **C**. Representative Western blot analysis and the summarized results (mean ± SD, n=3) of MCL-1 protein half-life in BEAS-2B-As+BaP cells treated with 5 µM of cycloheximide (Chx) for 0, 20, 40, 60, or 80 minutes with or without Wortmannin pre-treatment (2 µM, 2 h). * *p*<0.05 compared to Wortmannin pre-treatment groups. **D.** Representative Western blot analysis of MCl-1 IP experiment in BEAS-2B-As+BaP cell treated with a vehicle control or Wortmannin (2 µM, 24 h). IP: immunoprecipitation; IB: immunoblotting; WCL: whole cell lysate. **E.** Representative Western blot analysis of MCL-1 protein level in BEAS-2B-As+BaP cells pre-treated with a vehicle control or with different concentrations of Wortmannin for 24 h, followed by a vehicle control or MG132 (10 µM, 2 h) treatment. **F.** Representative Western blot analysis of phosphor-Akt and total Akt levels in the vector control and Myr-Akt1 stable overexpression BEAS-2B-As+BaP cells. **G.** Representative Western blot analysis of MCL-1, phosphor-Akt and total Akt protein levels in the vector control and Myr-Akt1 stable overexpression BEAS-2B-As+BaP cells treated with different concentrations of Wortmannin for 24 h. **H.** Representative Western blot analysis of total and cleaved PARP and caspase-3 protein levels in the vector control and Myr-Akt1 stable overexpression BEAS-2B-As+BaP cells treated with 20 µM of ABT-737 with or without 2 µM of Wortmannin co-treatment for 24 h. Similar results were obtained in the repeated experiments.

We next explored the mechanism of how MCL-1 protein stability is increased in the co-exposure-transformed cells. As mentioned above, our recent study showed that the co-exposure-transformed BEAS-2B cells have a significantly higher level of Akt activation than the arsenic or BaP exposure alone-transformed cells 14. Similarly, the highest Akt activation levels are also detected in arsenic and BaP co-exposure-transformed 16HBE cells (Figure S2). Since Akt pathway activation is usually linked with cell survival and apoptosis resistance 25, we first determined whether inhibition of Akt reduces MCL-1 protein stability. As expected, the PI3K inhibitor Wortmannin treatment greatly and dose-dependently decreases the level of phosphor-Akt (the activated Akt) in the co-exposure-transformed cells (Figure 3A). However, the Wortmannin treatment had no significant effect on the mRNA level of MCL-1 (Figure 3B), indicating that inhibition of the PI3K/Akt pathway has no effect on MCL-1 gene transcription. In contrast, when cells were treated with the protein synthesis inhibitor Chx and Wortmannin together, the MCL-1 protein half-life was significantly reduced (Figure 3C). The IP experiment showed that Wortmannin treatment greatly increases the MCL-1 protein ubiquitination level (Figure 3D), which will increase its proteasome degradation. Indeed, the proteasome degradation inhibitor MG132 treatment completely prevented the decrease of MCL-1 protein level caused by the Wortmannin treatment (Figure 3E). Together, these results suggest that the high PI3K/Akt activity in co-exposure-transformed cells plays an important role in maintaining MCL-1 protein stability, likely by reducing MCL-1 protein ubiquitination and subsequent proteasome degradation.

To further demonstrate the role of Akt activity in regulating MCL-1 protein levels and apoptosis resistance, we stably expressed a constitutive active Akt mutant (Myr-Akt1) in the co-exposure-transformed cells. Successful overexpression of the constitutively active Akt is shown in Figure 3F. The PI3K inhibitor Wortmannin treatment could not reduce MCL-1 protein levels in the Myr-Akt1 overexpression cells (Figure 3G), confirming the important role of Akt activity in maintaining MCL-1 protein levels. In consistence with the apoptotic resistance results shown in Figure 1A, ABT-737 treatment only weakly induced apoptosis in co-exposure-transformed vector control cells (BEAS-2B-As+BaP-pLenti7.3) and induced no obvious apoptosis in the constitutively active Akt mutant stably expression cells (BEAS-2B-As+BaP-pLenti7.3-Myr-Akt1) (Figure 3H). However, when the vector control cells were treated with ABT-737 plus Wortmannin, a much stronger apoptosis was detected as evidenced by the increased levels of cleaved PARP and caspase-3 (Figure 3H). In contrast, the constitutive active Akt mutant stably expression cells remain to be resistant to ABT-737 plus Wortmannin treatment (Figure 3H). Collectively, these results indicate that the higher Akt activation in the co-exposure-transformed cells increases MCL-1 protein stability and apoptosis resistance.

### Inhibition of the mammalian target of rapamycin (mTOR) significantly reduces MCL-1 protein stability in arsenic and BaP co-exposure-transformed cells and reverses their apoptosis resistance

**Figure 4:**
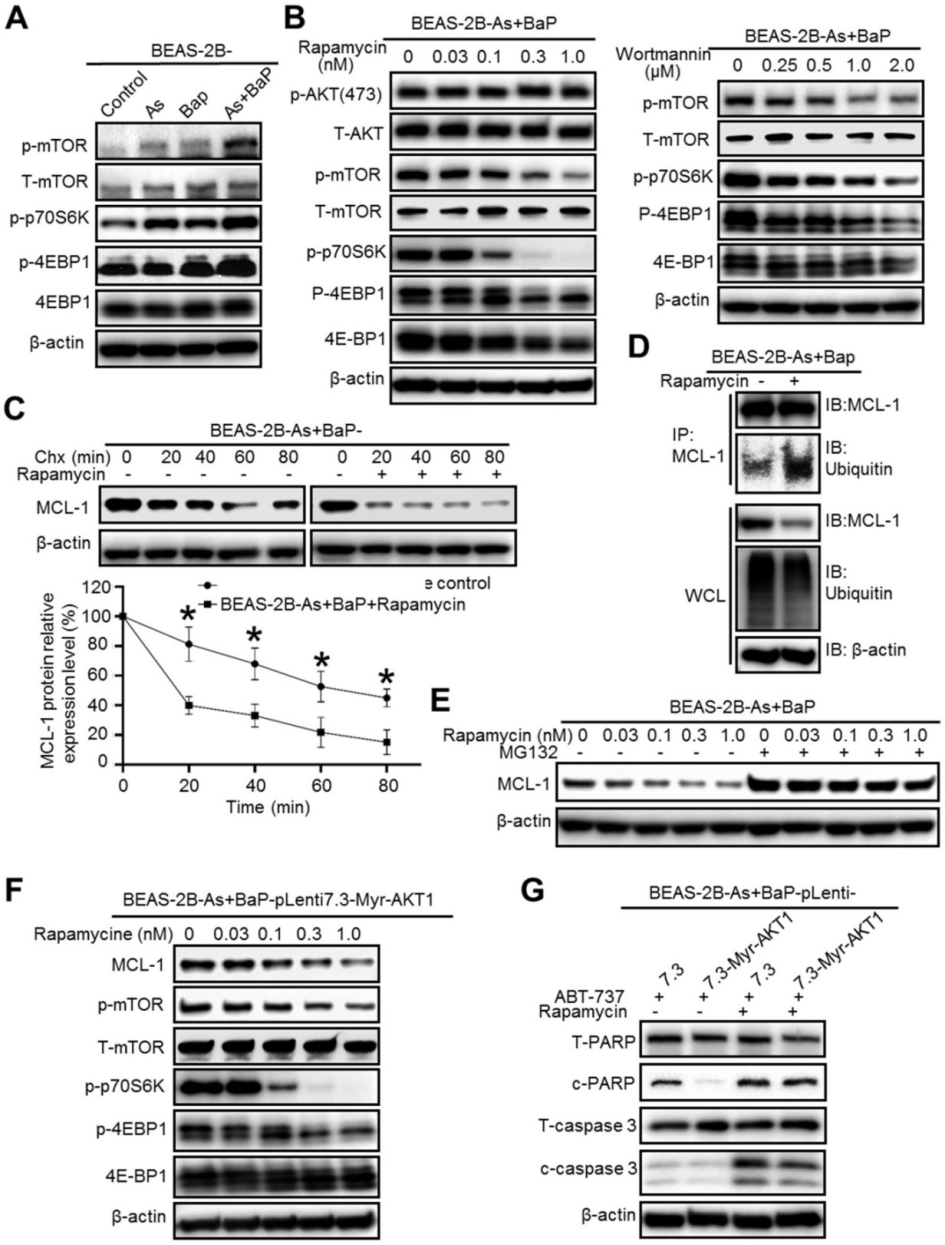
Inhibition of the mTOR pathway significantly reduces MCL-1 protein stability in arsenic and BaP co-exposure-transformed cells and reverses their apoptosis resistance. **A.** Representative Western blot analysis of the mTOR pathway protein phosphorylation levels in the BEAS-2B-Control, BEAS-2B-As, BEAS-2B-BaP and BEAS-2B-As+BaP cells. **B.** Representative western blot analysis of the effects of different concentrations of Wortmannin (0.25, 0.5, 1.0, 2.0 µM) or rapamycin (0.03, 0.1, 0.3, 1.0 nM) treatment (24 h) on the Akt/mTOR pathway protein phosphorylation levels in BEAS-2B-As+BaP cells. **C.** Representative Western blot analysis and the quantitated results (mean ± SD, n=3) of MCL-1 protein half-life in BEAS-2B-As+BaP cells treated with 5 µM of cycloheximide (Chx) for 0, 20, 40, 60, or 80 minutes with or without rapamycin pre-treatment (1 nM, 2 h). *p<0.05 compared to rapamycin pre-treatment groups. **D.** Representative Western blot analysis of MCl-1 IP experiment in BEAS-2B-As+BaP cell treated with a vehicle control or rapamycin (1 nM, 24 h). IP: immunoprecipitation; IB: immunoblotting; WCL: whole cell lysate. **E.** Representative Western blot analysis of MCL-1 protein level in BEAS-2B-As+BaP cells pre-treated with a vehicle control or different concentrations of rapamycin for 24 h, followed by a vehicle control or MG132 (10 µM, 2 h) treatment. **F.** Representative Western blot analysis of MCL-1 protein levels and mTOR pathway protein phosphorylation levels in Myr-Akt1 stable overexpression BEAS-2B-As+BaP cells treated with different concentrations of rapamycin for 24 h. **G.** Representative Western blot analysis of total and cleaved PARP and caspase-3 protein levels in the vector control and Myr-Akt1 stable overexpression BEAS-2B-As+BaP cells treated with 20 µM of ABT-737 with or without 1 nM of rapamycin co-treatment for 24 h. Similar results were obtained in the repeated experiments.

We next wanted to determine the molecular target down-stream of Akt that regulates MCL-1 protein stability. The mTOR is one of the well-studied down-stream targets of Akt and many studies have shown that the PI3K/Akt/mTOR pathway plays important roles in cancer, particularly cancer cell survival, growth and apoptosis resistance 29. Given the fact that Akt is highly activated in the co-exposure-transformed cells 14, we expected that the mTOR pathway is also highly activated in these cells. Indeed, Western blot analysis revealed that the phosphor-levels of mTOR and its down-stream targets p70 S6 kinase (p70S6K) and 4E-BP1 are drastically higher in the co-exposure-transformed cells than that in control, arsenic or BaP exposure alone-transformed cells (Figure 4A), indicating the high activation of the mTOR pathway. Moreover, inhibition of Akt by the Wortmannin treatment greatly and dose-dependently reduced the phosphor-levels of mTOR, p70S6K and 4E-BP1 (Figure 4B). However, inhibition of mTOR with rapamycin greatly and dose-dependently reduced the phosphor-levels of mTOR, p70S6K and 4E-BP1 but had no effect on the phosphor-level of Akt (Figure 4B), indicating that the mTOR pathway is activated down-stream of the PI3K/Akt pathway. In consistent with the highest Akt activation levels detected in arsenic and BaP co-exposure-transformed 16HBE cells, further Western blot analysis also revealed that phosphor-levels of mTOR and its down-stream targets p70S6K and 4E-BP1 are significantly higher in the co-exposure-transformed 16HBE cells than that in control, arsenic or BaP exposure alone-transformed 16HBE cells (Figure S2).

To determine whether the mTOR pathway plays a role in stabilizing MCL-1 protein in the co-exposure-transformed cells, we treated cells with Chx and rapamycin. It was found that rapamycin treatment significantly shortened MCL-1 protein half-life (Figure 4C). IP experiments showed that rapamycin treatment greatly increases the level of MCL-1 protein ubiquitination although the overall cellular protein ubiquitination level is even slightly reduced (Figure 4D). Furthermore, the proteasome degradation inhibitor MG-132 treatment completely prevented the decrease of MCL-1 protein levels caused by the rapamycin treatment (Figure 4E). Together, these results suggest that the highly activated-mTOR pathway in the co-exposure-transformed cells increases MCL-1 protein stability, likely by reducing MCL-1 protein ubiquitination and subsequent proteasome degradation.

Since mTOR is activated down-stream of Akt in the co-exposure-transformed cells (Figure 4B), we expect that inhibition of mTOR could reduce MCL-1 protein levels and sensitize the constitutively active Akt stable expressing cells (BEAS-2B-As+BaP-Myr-Akt1) to ABT-737-induced apoptosis. Indeed, Western blot analysis confirmed that the rapamycin treatment dose-dependently reduces the phosphor-levels of mTOR, p70S6K, 4E-BP1 and MCL-1 as well in BEAS-2B-As+BaP-Myr-Akt1 cells (Figure 4F).

As a result, the rapamycin treatment was capable of inducing apoptosis in the vector control and the Myr-Akt1 stably expressing cells to a similar extent (Figure 4G).

### The deubiquitinase USP7 is a critical target of the mTOR pathway and reduces MCL-1 protein ubiquitination level, preventing MCL-1 proteasome degradation

**Figure 5:**
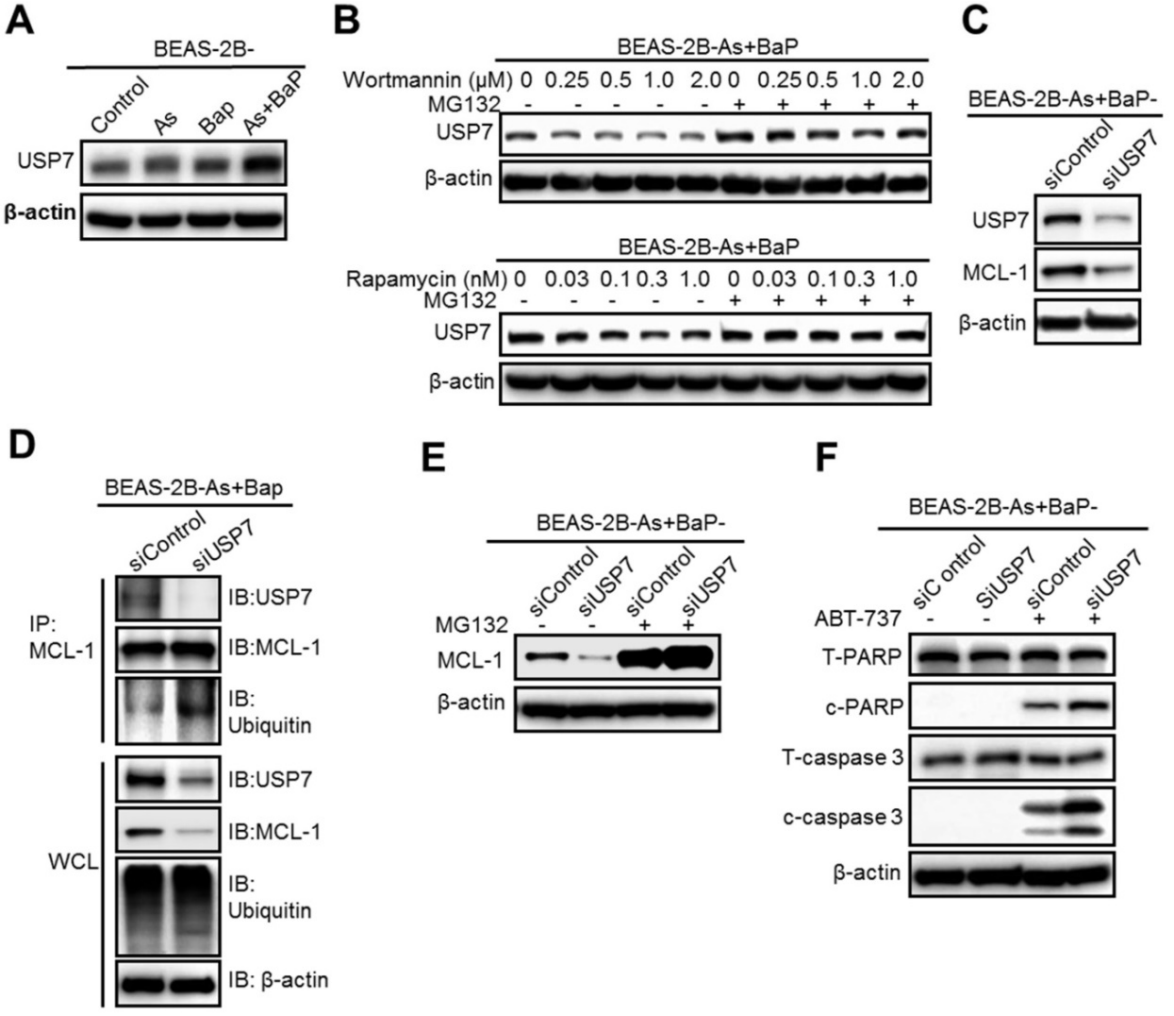
The deubiquitinase USP7 is a critical target of the mTOR pathway and reduces MCL-1 protein ubiquitination level, preventing MCL-1 proteasome degradation. **A.** Representative Western blot analysis of USP7 levels in BEAS-2B-Control, BEAS-2B-As, BEAS-2B-BaP and BEAS-2B-As+BaP cells. **B.** Representative Western blot analysis of USP7 protein levels in BEAS-2B-As+BaP cells pre-treated with different concentrations of Wortmannin or rapamycin for 24 h, followed by a vehicle control or 10 µM of MG132 co-treatment for 2 h. **C.** Representative Western blot analysis of USP7 and MCL-1 protein levels in BEAS-2B-As+BaP cells transfected with control siRNA (siControl) or USP7 siRNA (siUSP7) oligoes for 48 h. **D.** Representative Western blot analysis of MCL-1 IP experiment in BEAS-2B-As+BaP cells transfected with control siRNA (siControl) or USP7 siRNA (siUSP7) oligoes for 48 h. IP: immunoprecipitation; IB: immunoblotting; WCL: whole cell lysate. **E.** Representative Western blot analysis of MCL-1 protein levels in BEAS-2B-As+BaP cells transfected with control siRNA (siControl) or USP7 siRNA (siUSP7) oligoes for 48 h , followed by a vehicle control or 10 µM of MG132 co-treatment for 2 h. **F.** Representative Western blot analysis of total and cleaved PARP and caspase-3 protein levels in BEAS-2B-As+BaP cells transfected with control siRNA (siControl) or USP7 siRNA (siUSP7) oligoes for 48 h, followed by a vehicle control or 20 µM of ABT-737 co-treatment for 24 h. Similar results were obtained in the repeated experiments.

The PI3K/Akt/mTOR pathway is critical in regulating protein levels 30, 31. Our above results showed that inhibition of the PI3K/Akt/mTOR pathway, either by wortmannin or rapamycin treatment, greatly increases the level of MCL-1 protein ubiquitination, but does not significantly increase overall cellular protein ubiquitination levels (Figure 3D and Figure 4D). These findings suggest that the PI3K/Akt/mTOR pathway increases the level of one or several protein deubiquitinases that deubiquitinize MCL-1 and increase MCL-1 protein stability in the co-exposure-transformed cells. We then screened and compared the protein levels of 10 deubiquitinases and found that the levels of the deubiquitinase USP7 (also known as Herpesvirus-associated ubiquitin-specific protease, HAUSP), USP10 and USP18 are significantly higher in the co-exposure-transformed cells than that in the control, arsenic or BaP exposure alone-transformed cells (Figure 5A, Figure S3A). Wortmannin or rapamycin treatment dose-dependently reduced USP7 protein levels but had no effect on USP10 and USP18 levels (Figure 5B, Figure S3B). Moreover, wortmannin or rapamycin co-treatment with the proteasome degradation inhibitor MG132 recovered USP7 protein levels (Figure 5B).

We then focused on USP7 and next determined whether USP7 plays a role in regulating MCL-1 protein levels in the co-exposure-transformed cells. First, it was found that siRNA knocking down USP7 levels greatly reduces MCL-1 protein levels (Figure 5C). Second, siRNA knocking down USP7 levels significantly increased the level of MCL-1 protein ubiquitination (Figure 5D). Third, treatment with the proteasome degradation inhibitor MG132 greatly recovered MCL-1 protein levels in cells transfected with the USP7 siRNA (Figure 5E). Together, these results suggest that USP7 plays an important role in maintaining MCL-1 protein levels in the co-exposure-transformed cells, likely by reducing MCL-1 protein ubiquitination and preventing its proteasome degradation.

In consistence with its critical role in maintaining MCL-1 protein level, siRNA knocking down USP7 levels increased ABT-737 treatment-induced apoptosis in the co-exposure-transformed cells (Figure 5F). The quantitated results of Western blots in Figure 5F are shown in Figure S4A-B. Moreover, the effect of USP7 knockdown on ABT-737 treatment-induced apoptosis was further determined using the flow cytometry-based analysis of Annexin V-positive cells. Similarly, it was found that USP7 knockdown significantly increased apoptosis (19.51% ± 0.699 in ABT-737-treated control siRNA-transfected cells vs 31.34% ± 0.1817 in ABT-737-treated USP7-transfected cells) (Figure S4C-D).

### Stably expressing MCL-1 prevents wortmannin or rapamycin plus ABT-737 treatment from inducing apoptosis in arsenic and BaP co-exposure-transformed cells

**Figure 6:**
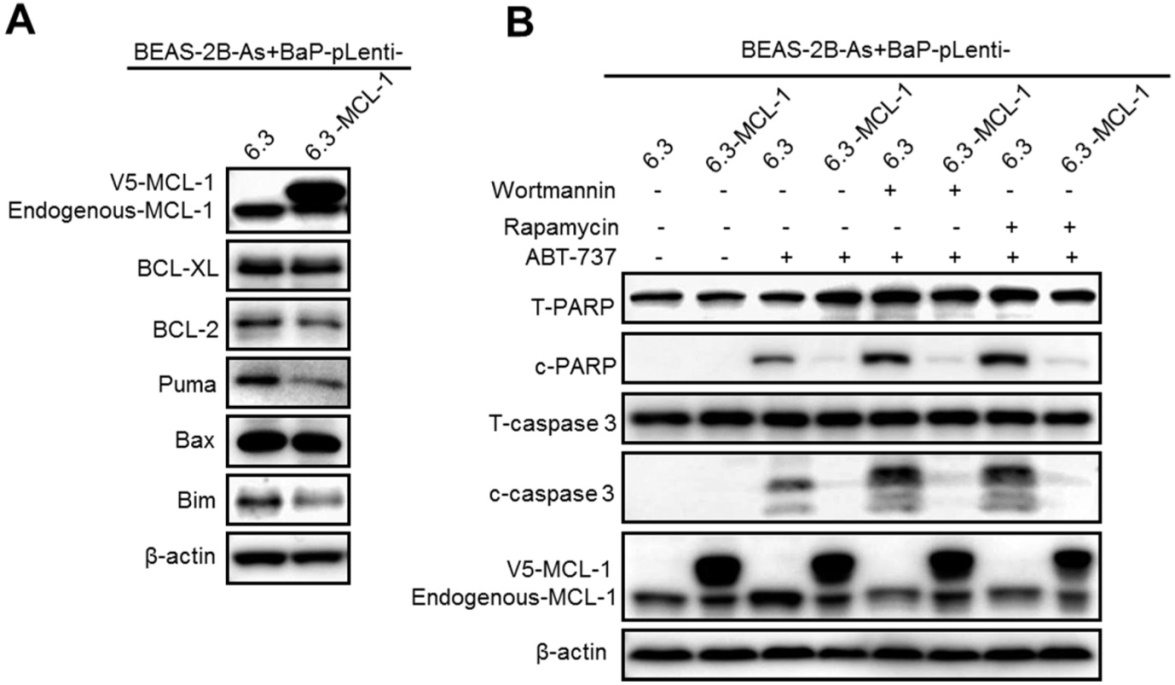
Stably expressing MCL-1 prevents Wortmannin or rapamycin plus ABT-737 treatment from inducing apoptosis in arsenic and BaP co-exposure-transformed cells. **A.** Representative Western blot analysis of MCL-1, BCL-XL, BCL-2, Puma, Bax and Bim protein levels in vector control and MCL-1 stably overexpressing BEAS-2B-As+BaP cells. **B.** Representative Western blot analysis of total and cleaved PARP and caspase-3 protein levels in vector control and MCL-1 stably overexpressing BEAS-2B-As+BaP cells treated with a vehicle control, Wortmannin (2 µM), or rapamycin (1 nM) plus 20 µM of ABT-737 for 24 h. Similar results were obtained in the repeated experiments.

To further demonstrate that the PI3K/Akt/mTOR pathway activation mediates apoptosis resistance by up-regulating MCL-1 expression levels in the co-exposure-transformed cells, we generated vector control and MCL-1 stably overexpressing cells (Figure 6A). Stably overexpressing MCL-1 in the co-exposure-transformed cells did not significantly change the protein levels of BCL-XL, BCL-2 and Bax, although it reduced the protein levels of Puma and Bim (Figure 6A). In consistence with the observed apoptosis resistance in the co-exposure-transformed cells shown in Figure 1B-D, ABT-737 treatment caused weak apoptosis in the vector control cells but no apoptosis in MCL-1 overexpressing cells (Figure 6B). Moreover, inhibition of the PI3K/Akt activity by wortmannin or inhibition of the mTOR activity by rapamycin enhanced ABT-737 treatment-induced apoptosis in the vector control cells (Figure 6B). In contrast, inhibition of the PI3K/Akt activity by wortmannin or inhibition of the mTOR activity by rapamycin plus ABT-737 treatment failed to cause apoptosis in MCL-1 stably expressing cells (Figure 6B). Together, these results indicate that the PI3K/Akt/mTOR pathway activation mediates apoptosis resistance by up-regulating MCL-1 expression levels in the co-exposure-transformed cells.

### Stably knocking down MCL-1 expression levels in arsenic and BaP co-exposure- transformed cells significantly reduces their CSC-like property and tumorigenicity

**Figure 7:**
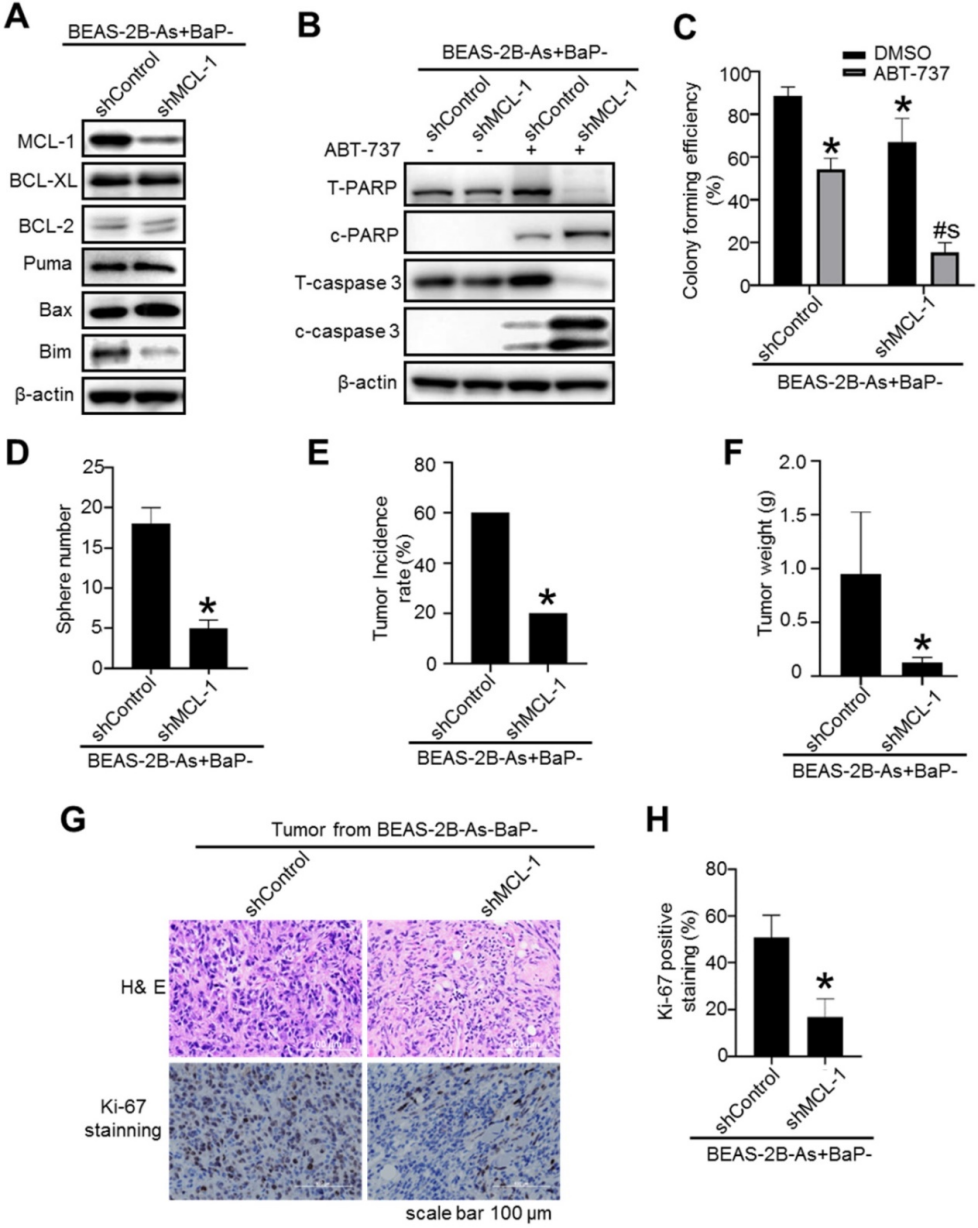
Stably knocking down MCL-1 expression levels in arsenic and BaP co-exposure- transformed cells significantly reduces their apoptosis resistance, CSC-like property and tumorigenicity. **A.** Representative Western blot analysis of MCL-1, BCL-XL, BCL-2, Puma, Bax and Bim protein levels in shRNA vector control (shControl) and MCL-1 stably knocked down (shMCL-1) BEAS-2B-As+BaP cells. **B.** Representative Western blot analysis of total and cleaved PARP and caspase-3 protein levels in vector control (shControl) and MCL-1 stably knocked down (shMCL-1) BEAS-2B-As+BaP cell streated with 20 µM of ABT-737 for 24 h. **C-D.** Effect of stably knocking down MCL-1 expression in arsenic and BaP co-exposure transformed cells on their capability of clonal growth (**C**) and forming suspension culture spheres (**D**) (mean ± SD, n=3). *p<0.05, compared to BEAS-2B-As+BaP-shControl cells; # *p*<0.05, compared to BEAS-2B-As+BaP-shControl cells treated with ABT-737; $ *p*<0.05, compared to BEAS-2B-As+BaP-shMCL-1 cells treated with ABT-737. Similar results were obtained in the repeated experiments. **E-H.** Mouse xenograft tumor incidence rate (**E**), tumor weight (mean + SD, n=4-12) (**F**), and representative tumor tissue section H&E and Ki-67 (**G**) IHC staining and the quantitated results (**H**) of Ki-67 IHC staining. *p< 0.05, compared to tumors from injection of shRNA control cells. Ki67 positive cells (brown) were counted in 30 randomly selected fields (20×) per mouse tumor tissue section. Four mouse tumor tissues from each group were counted. The quantitated results are presented as percent of cells with Ki-67 positive staining per field of view per mouse (mean + SD, n=4). *p< 0.05, compared to tumors from injection of shRNA control cells.

To further determine the role of MCL-1 in maintaining the malignant phenotypes of the co-exposure-transformed cells, we generated a shRNA vector control and MCL-1 stable knockdown cells (Figure 7A). A significant knockdown of MCL-1 protein levels in the co-exposure-transformed cells is shown in Figure 7A. Stably knocking down MCL-1 levels in the co-exposure-transformed cells did not significantly change the protein levels of BCL-XL, BCL-2, Bax and Puma, although it reduced the protein level of Bim (Figure 7A). In consistence with the apoptotic enhancing effect of transiently knocking down MCL-1 levels using siRNA shown in Figure 1H, stably knocking down MCL-1 levels greatly increased ABT-737 treatment-induced apoptosis as evidenced by the diminished total PARP and caspase-3 protein levels and increased cleaved PARP and caspase-3 protein levels (Figure 7B). Similarly, stably knocking down MCL-1 also significantly reduced the clonal growth of the co-exposure-transformed cells with or without ABT-737 treatment (Figure 7C).

Our recent study showed that the co-exposure-transformed cells display a strong CSC-like property and tumorigenicity 14. Further Western blot analysis showed that the co-exposure-transformed cells have the highest protein levels of key CSC marker KLF4 and KLF5 (Figure S5A). Knocking known MCL-1 levels in the co-exposure-transformed cell greatly decreased their KLF5 protein levels (Figure S5B) and their CSC-like property as determined by the ALDEFLUOR assay (Figure S5C) and the suspension culture sphere formation assay (Figure 7D). These results suggest that MCL-1 knockdown reduces the co-exposure-transformed cells' CSC-like property. This was further confirmed by the subsequent nude mouse tumorigenesis experiment. It was found that injection of MCL-1 stable knockdown cells produces significantly less and smaller xenograft tumors in nude mice compared to the injection of the shRNA control cells (Figure 7E-F). The tumor incidence rate in mice injected with MCL-1 stable knockdown cells was 20%, however, the tumor incidence rate in mice injected with shRNA control cells was 60% (*p*<0.05). Further tumor tissue analysis showed that the positive staining of the proliferation marker Ki-67 is significantly less in the tumors from injection of MCL-1 knockdown cells, compared to the tumors from injection of shRNA control cells (Figure 7G-H). In contrast, knocking down BCL-XL levels in the co-exposure-transformed cells did not reduce their key CSC marker KLF4 and KLF5 protein levels (Figure S6A). Although knocking down BCL-XL levels in the co-exposure-transformed cells slightly reduced the percentage of ALDEFLUOR positive cells (Figure S5C), it had no effect on their suspension culture formation capability (Figure S6B). Together, these results indicate that MCL-1 up-regulation but not the BCL-XL up-regulation in the co-exposure-transformed cells plays an important role in mediating their apoptosis resistance, CSC-like property and tumorigenesis.

## Discussion

**Figure 8:**
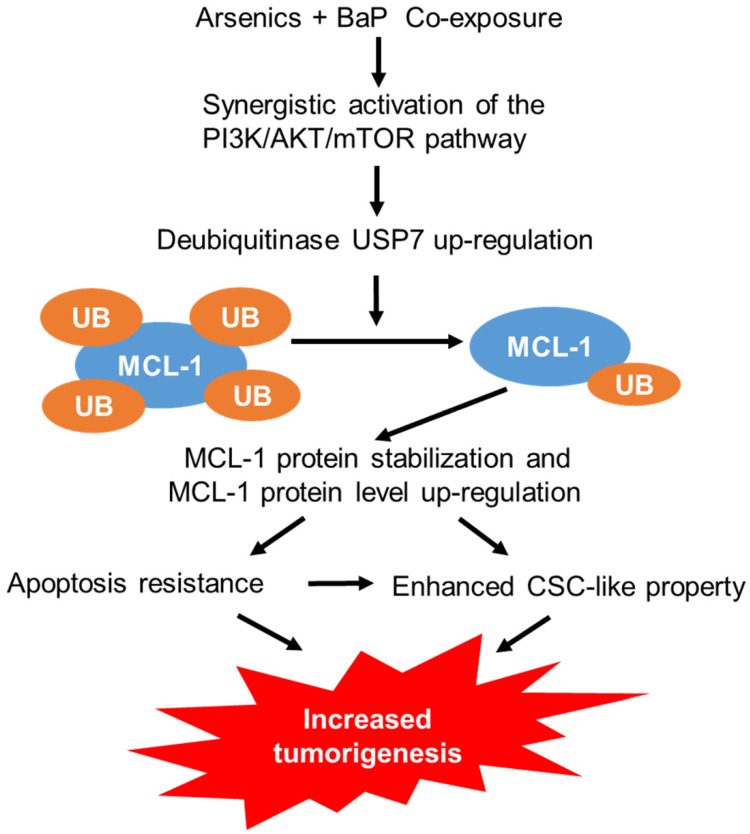
A schematic description on the mechanism of arsenic and BaP co-exposure enhancing CSC-like property and tumorigenesis.

While humans are often exposed to mixtures of environmental pollutants, our understanding of the health effects and the mechanism of mixture exposures is limited. Current studies on the mechanism of toxic effects of environmental pollutants primarily focus on single pollutant exposures. However, studies have shown that the adverse health effects and the underlying mechanism of mixture exposures could be significantly different from that of single exposures. For example, our recent and others' previous studies showed that arsenic and BaP co-exposure causes significantly stronger effects than arsenic or BaP exposure alone in inducing cell malignant transformation, CSC-like property and tumorigenesis 12-14, 32, although the underlying mechanisms are not well understood. In this study, we provide evidence demonstrating that arsenic and BaP co-exposure causes a stronger activation of the PI3K/Akt/mTOR pathway to increase the expression level of a deubiquitinase USP7, which in turn up-regulates the level of an anti-apoptotic protein MCL-1 by reducing its ubiquitination and subsequent proteasome degradation. The up-regulation of MCL-1 protein levels plays a critical role in arsenic and baP co-exposure-enhanced CSC-like property and tumorigenesis (Figure 8). This study provides the first evidence showing that the anti-apoptotic MCL-1 protein stability is regulated by the deubiquitinase USP7 in tumor cells (arsenic and BaP co-exposure-transformed cells). The findings from this study not only offer a new mechanistic insight for the synergistic tumorigenic effect of arsenic and BaP co-exposure, but also reveal an important role and a new mechanism for MCL-1 protein up-regulation in the tumorigenic process.

Normal tissue homeostasis is maintained by the well-controlled and balanced cell growth and death. Cancer develops from uncontrolled, accelerated cell growth and reduced cell death or apoptosis resistance 33. Mechanistically, apoptosis resistance may result from increased levels of cellular anti-apoptotic proteins and/or reduced levels of pro-apoptotic proteins. By using a series of biochemical, pharmacological and genetic approaches, we not only demonstrated in this study that MCL-1 up-regulation mediates apoptosis resistance in arsenic and BaP co-exposure-transformed cells, but also showed that down-regulating MCL-1 protein levels in the co-exposure-transformed cells significantly reduces their CSC-like property and tumorigenicity. Previous studies showed that down-regulating MCL-1 protein levels sensitizes lung cancer cells to ABT-737-induced apoptosis 34, 35. Our above findings are thus important, not only because they indicate that MCL-1 up-regulation plays a critical role in arsenic and BaP co-exposure-induced tumorigenesis, but also because they suggest that strategies of targeting MCL-1 could be effective in preventing and treating lung cancer resulting from arsenic and BaP co-exposure.

Understanding the mechanism of how arsenic and BaP co-exposure up-regulates MCL-1 protein levels may identify additional targets to prevent and treat arsenic and BaP co-exposure-caused lung cancer. MCL-1 is different from other anti-apoptotic proteins of the BCL-2 family in that its protein level is quickly turned over via the action of ubiquitin ligases and deubiquitinases 5-7. The high activity of glycogen synthase kinase-3 (GSK-3) plays a key role in phosphorylating MCL-1 protein to prime it for ubiquitination and subsequent proteasome degradation 6. Since GSK-3 is a well-established down-stream target of Akt 36, 37 and our recent study showed that arsenic and BaP co-exposure causes a significantly stronger activation of Akt 14, we first analyzed and compared GSK-3 activity among the control, arsenic or BaP exposure alone-transformed cells and the co-exposure-transformed cell. However, we failed to observe any correlation between the GSK-3 activity levels and MCL-1 protein levels among the four groups of cells, suggesting that GSK-3 does not play a major role in regulating MCL-1 protein levels in our cell models. We then began to analyze mTOR, another well-known down-stream target of Akt 36 and found that a stronger activation of Akt leads to a stronger activation of the mTOR pathway in the co-exposure-transformed cells. We further demonstrated that the highly activated mTOR pathway is critical for the up-regulation of MCL-1 protein levels. Although the mTOR pathway is well known for regulating protein translation 31, 38, our findings show that the mTOR pathway activation also plays an important role in regulating MCL-1 protein stability. This statement is supported by the findings which show that the decrease of MCL-1 protein levels resulting from the mTOR pathway inhibitor rapamycin treatment could be reversed by the proteasome degradation inhibitor MG-132 treatment. We further demonstrated that the mTOR pathway activation increases MCL-1 protein stability by up-regulating the protein level of the deubiquitinase USP7, which reduces MCL-1 protein ubiquitination and its subsequent proteasome degradation. Previous studies showed that two deubiquitinases, USP9X and USP13, stabilize MCL-1 protein levels and promote tumor growth 5, 7. The findings from this study provide the first evidence to show that the deubiquitinase USP7 could also stabilize MCL-1 protein levels. Collectively, the findings from this study suggest that mTOR and USP7 may serve as additional novel targets for developing strategies to prevent and treat arsenic and BaP co-exposure-caused lung cancer.

Previous studies also showed that MCL-1 is essential for the normal stem cell and cancer stem cell (CSC) self-renewal 39, 40. This provides an explanation for the findings from this study showing that stably knocking down MCL-1 levels in the co-exposure-transformed cells significantly reduces their CSC-like property and tumorigenicity. CSCs are considered as cancer initiation cells to initiate tumor formation 41-43. Therefore, up-regulation of MCL-1 plays a critical role in arsenic and BaP co-exposure-induced synergistic lung tumorigenic effect by increasing CSCs or CSC-like cells. However, it remains to be determined whether MCL-1 up-regulation enhances CSC-like property through its effect on apoptosis resistance. Further studies are needed to investigate how MCL-1 up-regulation enhances arsenic and BaP co-exposure-induced CSC-like property. In addition, CSCs are also considered as a key mediator of developing cancer therapeutic resistance 44, 45. The critical role of MCL-1 in regulating CSC-like property thus provides additional rationale for targeting MCL-1 to treat lung cancer resulting from arsenic and BaP co-exposure.

In summary, this study demonstrates that MCL-1 protein levels are up-regulated in arsenic and BaP co-exposure-transformed cells, which plays an important role in arsenic and BaP co-exposure-induced CSC-like property and tumorigenesis. Moreover, this study also reveals for the first time that the deubiquitinase USP7 regulates MCL-1 protein stability.

## Supplementary Material

Supplementary figures.
