# Supplementary material for: Deubiquitinase USP7-mediated MCL-1 up-regulation enhances Arsenic and Benzo(a)pyrene co-exposure-induced Cancer Stem Cell-like property and Tumorigenesis
Source: Theranostics. 2020 Jul 11;10(20):9050–65. doi: 10.7150/thno.47897 (PMC7415806; doi:10.7150/thno.47897)
Supplement: Supplementary file 1 — Supplementary figures. [file thnov10p9050s1.pdf]

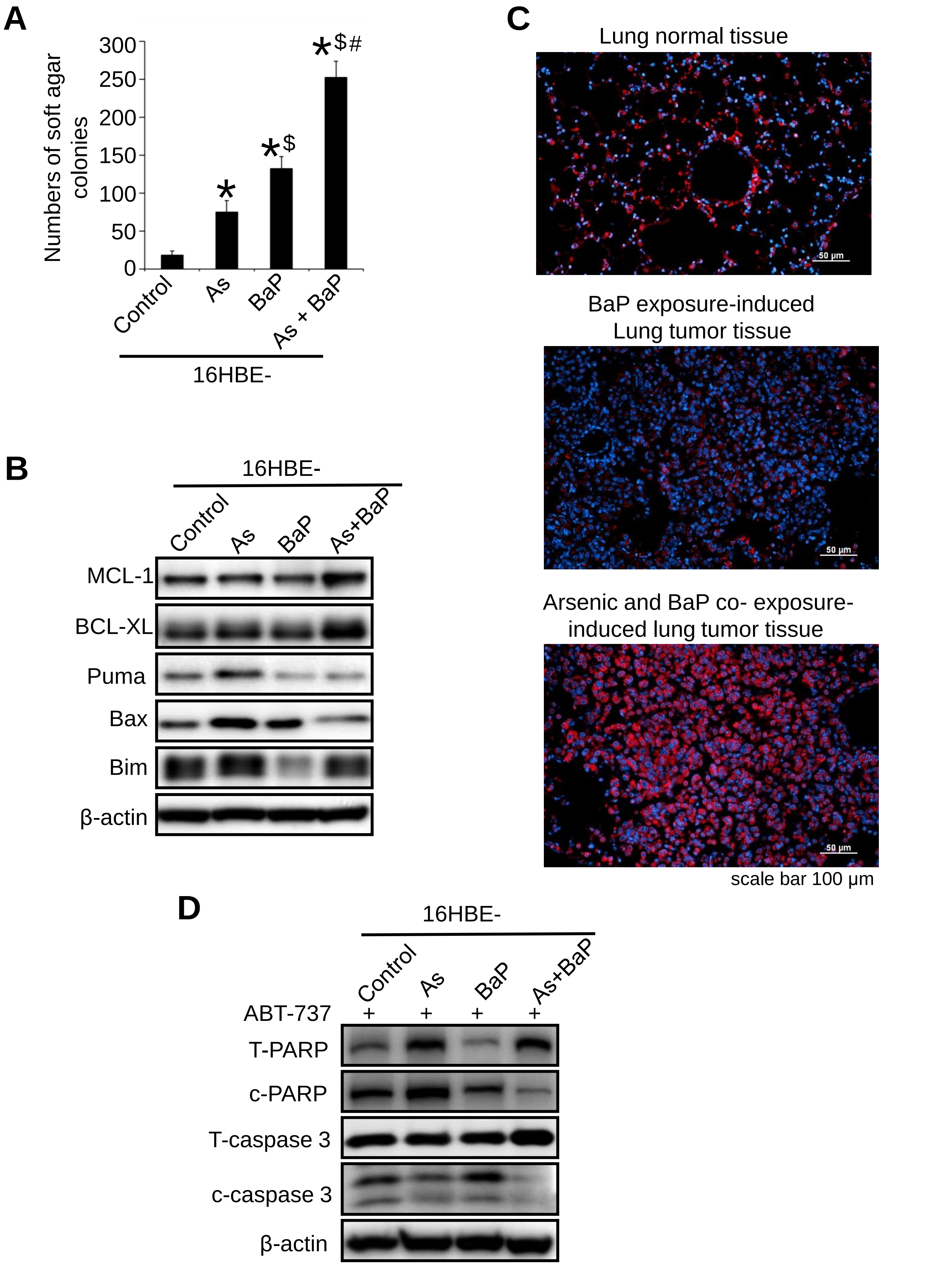
A
Numbers of soft agar colonies
300
250
200
150
100
50
0
*
*$
*$#
Control
As
BaP
As + BaP
16HBE-
B
16HBE-
Control
As
BaP
As+BaP
MCL-1
BCL-XL
Puma
Bax
Bim
β-actin
C
Lung normal tissue
50 μm
BaP exposure-induced
Lung tumor tissue
50 μm
Arsenic and BaP co- exposure-
induced lung tumor tissue
50 μm
scale bar 100 μm
D
16HBE-
Control
As
BaP
As+BaP
ABT-737
+
+
+
+
T-PARP
c-PARP
T-caspase 3
c-caspase 3
β-actin

Figure S1. MCL-1 protein levels are up-regulated in arsenic and BaP c-exposure-transformed 16HBE cells and mouse lung tumor tissues and the co-exposure-transformed 16HBE cells are resistant to ABT-737-induced apoptosis. **A.** Numbers of soft agar colonies formed by 16HBE cells chronically exposed to a vehicle control (DMSO), arsenic ($NaAsO_2$, 1 μM), BaP (2.5 μM), or arsenic ($NaAsO_2$, 1 μM) plus BaP (2.5 μM) for 40 weeks (mean ± SD, n=3). * p<0.05, compared to Control cells; $^{\$}$ p<0.05, compared to arsenic (As)-exposed cells; $^{\#}$ p<0.05, compared to BaP-exposed cells. **B.** Representative Western blot analysis of anti-apoptotic and pro-apoptotic protein levels in control, arsenic, BaP or arsenic plus BaP co-exposed 16HBE cells. **C.** Representative overlaid images of MCL-1 IF staining (red) and DNA DAPI staining (blue) in mouse lung normal tissue, BaP exposure alone-induced lung tumor tissue or arsenic plus BaP co-exposure-induced lung tumor tissue. Similar results were obtained in two additional mice In each group. Note: the red signals in normal lung tissues are mostly from red blood cells that do not have DNA DAPI blue staining, which are not MCL-1 positive staining. **D.** Representative Western blot analysis of total and cleaved PARP and caspase-3 protein levels in 16HBE cells treated with 10 μm of ABT-737 for 24 h.

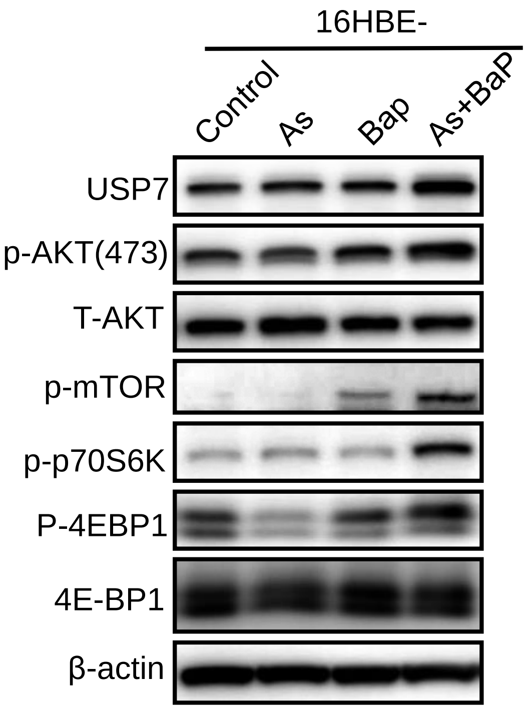

Figure S2. The Akt/mTOR pathway is highly activated and the USP7 protein levels are mostly up-regulated in arsenic and BaP co-exposure-transformed 16HBE cells. Representative Western blot analysis of USP7, phosphor-Akt, mTOR, p70S6K and 4EBP-1 protein levels in 16HBE cells exposed to a vehicle control (DMSO), arsenic ($NaAsO_2$, 1 μM), BaP (2.5 μM), or arsenic ($NaAsO_2$, 1 μM) plus BaP (2.5 μM) for 40 weeks.

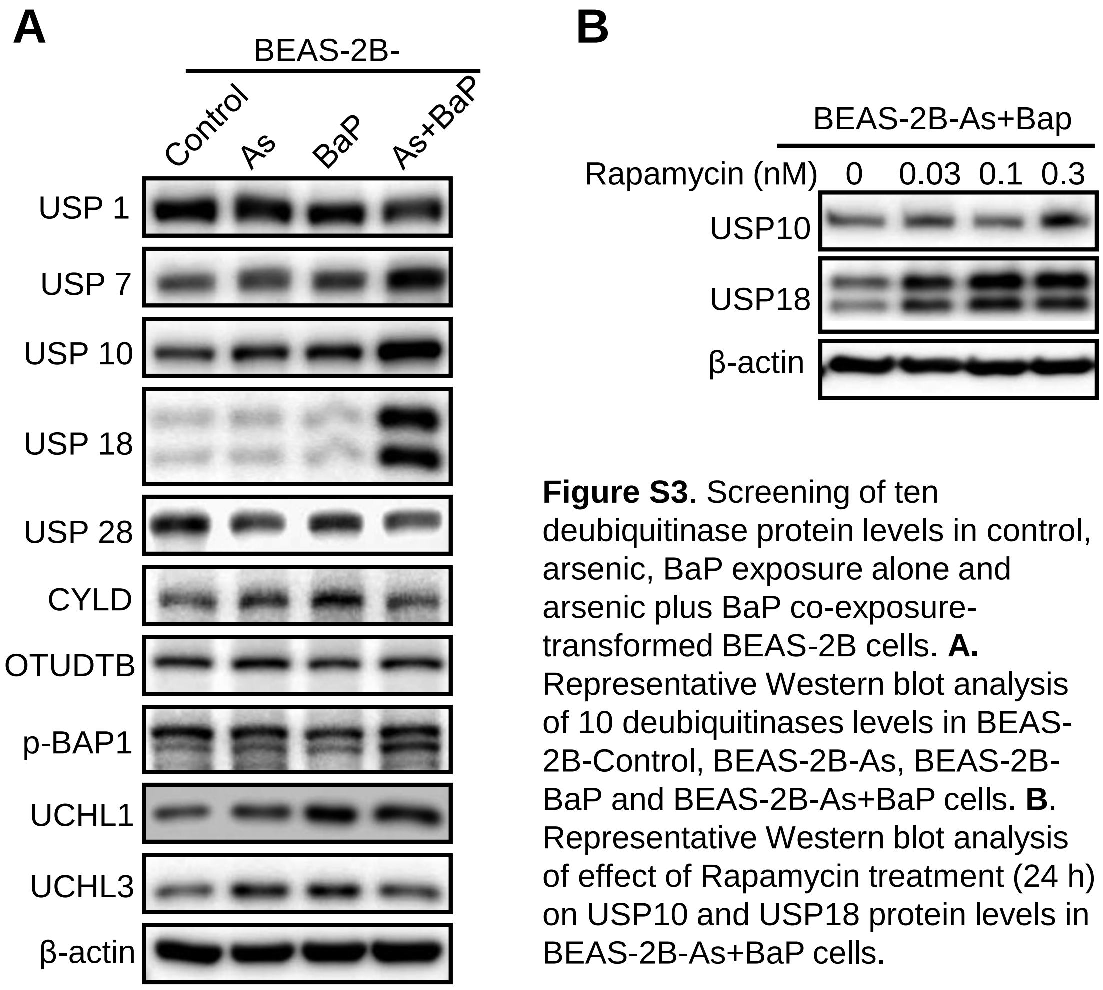

Figure S3. Screening of ten deubiquitinase protein levels in control, arsenic, BaP exposure alone and arsenic plus BaP co-exposure-transformed BEAS-2B cells. **A.** Representative Western blot analysis of 10 deubiquitinases levels in BEAS-2B-Control, BEAS-2B-As, BEAS-2B-BaP and BEAS-2B-As+BaP cells. **B**. Representative Western blot analysis of effect of Rapamycin treatment (24 h) on USP10 and USP18 protein levels in BEAS-2B-As+BaP cells.

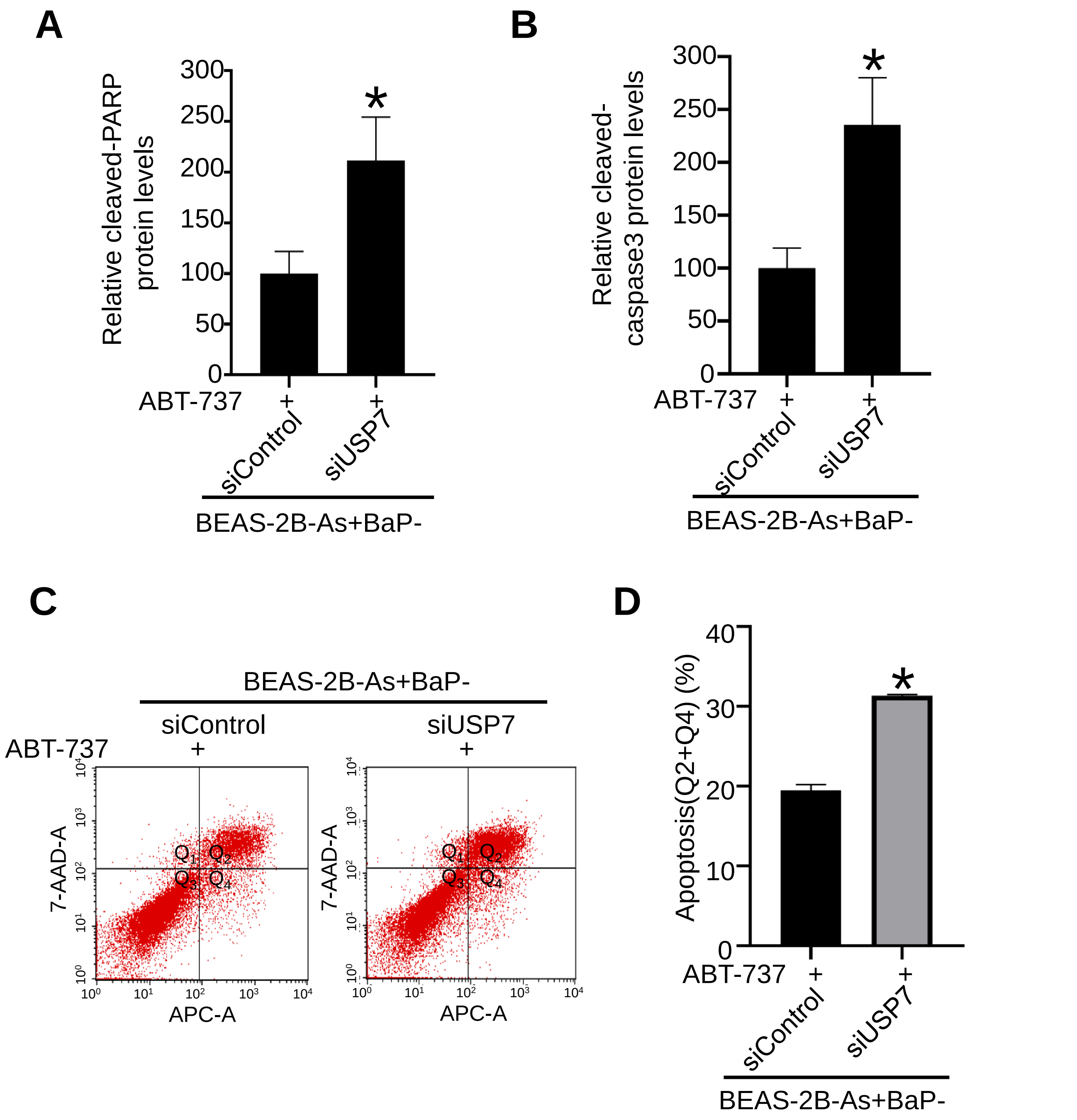

Figure S4. Quantitative analysis of effect of USP7 knockdown on ABT-737-induced apoptosis in arsenic and BaP co-exposure-transformed BEAS-2B cells. **A, B.** The ratio of cleaved PARP protein levels divided by the corresponding total PARP protein levels (**A**) and the ratio of cleaved caspse-3 protein levels divided by the corresponding total caspase-3 protein levels (**B**) in cells transfected with 100 nM of control siRNA or USP7 targeting siRNA and treated with 20 µM of ABT-737 for 24 h (mean $\pm$ SD, n=3). * $p<0.05$, compared to the BEAS-2B-As+BaP-siControl group. **C, D.** Representative histograms of flow cytometry analysis of apoptosis by Annexin V staining (**C**) and the quantitative results (mean $\pm$ SD, n=3) (**D**). Cells were treated in the same way as in panel A and B. Q1, Q2, Q3, Q4 indicate necrocytosis, late apoptosis cells, survival cells, and early apoptosis, respectively. *$p<0.05$, compared to the BEAS-2B-As+BaP-siControl group.

Supplementary Figure 5—Figure S5

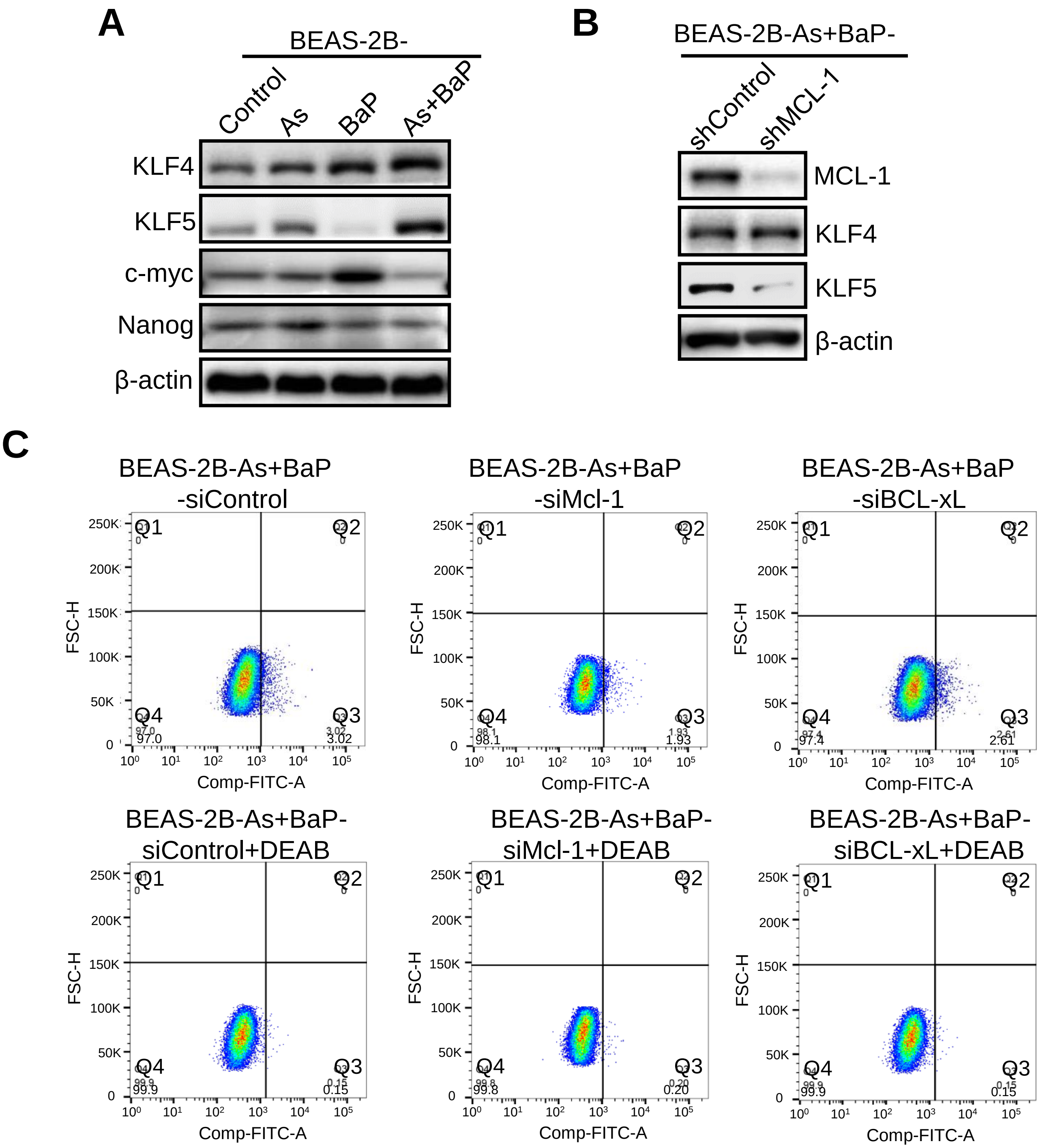

D

ALDEFLUOR positive cells (%) (Mean± SD, n=3)

		With DEAB pre-treatment
BEAS-2B siControl	2.9533±0.0611	0.29±0.1039
BEAS-2B siMcl-1	2.0167±0.0757*#	0.1767±0.02517
BEAS-2B siBCL-xL	2.51±0.0888*	0.1233±0.02309

Figure S5. Effect of knocking down MCL-1 or BCL-XL levels on cancer stem cell-like property of arsenic and BaP co-exposure-transformed BEAS-2B cells. **A**. Representative Western blot analysis of the expression levels of several cancer stem cell markers in passage-matched control cells, arsenic exposure alone-transformed cells, BaP exposure alone-transformed cells and arsenic plus BaP co-exposure-transformed cells. **B**. Representative Western blot analysis of KLF4 and KLF5 protein levels in shRNA vector control (shControl) and MCL-1 stably knocked down (shMCL-1) BEAS-2B-As+BaP cells. **C.** Representative histograms of flow cytometry ALDEFLUOR analysis of BEAS-2B-As+BaP cells transfected with 100 nM of Control siRNA (siControl) or MCL-1 siRNA (siMCL-1) or BCL-Xl siRNA (siBCL-XL) for 48 h. **D.** Quantification of ALDEFLUOR analysis (mean ± SD, n=3). * $p<0.05$, compare to BEAS-2B-As+BaP-siControl cells; $^{\#}$ $p<0.05$, compared to BEAS-2B-As+BaP-siBCL-Xl cells.

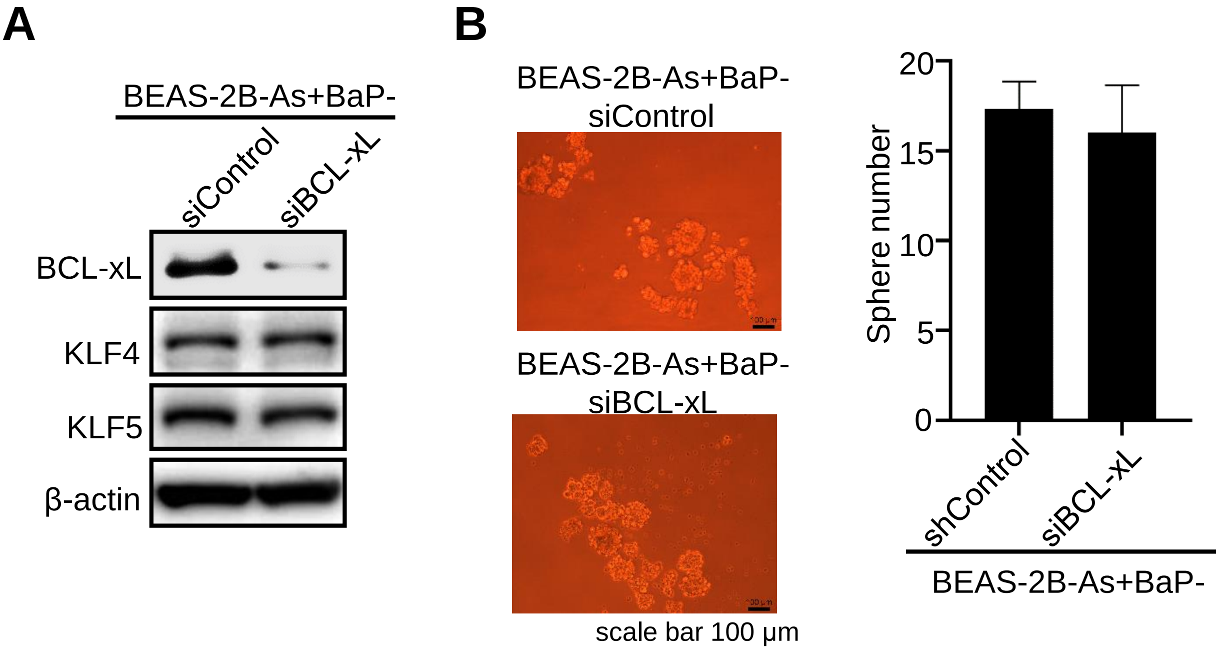

Figure S6. Effect of knocking down BCL-Xl levels in arsenic and BaP co-exposure-transformed BEAs-2B cells on their cancer stem cell property. **A.** Representative Western blot analysis of cancer stem cells marker KLF4 and KLF5 protein levels in BEAS-2B-As+BaP cells transfected with 100 nM of control siRNA (siControl) or BCL-xL targeting siRNA (siBCL-xL) for 48 h. **B.** Effect of knocking down BCL-xL expression in arsenic and BaP co-exposure transformed cells on their capability of forming suspension culture spheres (mean ± SD, n=3). BEAS-2B-As+BaP cells were transfected with 100 nM of control siRNA (siControl) or BCL-xL targeting siRNA (siBCL-xL). Forty-eight h after transfection, cells were collected for suspension culture sphere formation assay as described in Methods.
